# A novel mechanism for the biogenesis of outer membrane vesicles in Gram-negative bacteria

**DOI:** 10.1038/ncomms10515

**Published:** 2016-01-25

**Authors:** Sandro Roier, Franz G. Zingl, Fatih Cakar, Sanel Durakovic, Paul Kohl, Thomas O. Eichmann, Lisa Klug, Bernhard Gadermaier, Katharina Weinzerl, Ruth Prassl, Achim Lass, Günther Daum, Joachim Reidl, Mario F. Feldman, Stefan Schild

**Affiliations:** 1 Institute of Molecular Biosciences, University of Graz, NAWI Graz, BioTechMed-Graz, Humboldtstraße 50, A-8010 Graz, Austria; 2 Institute of Biochemistry, Graz University of Technology, NAWI Graz, BioTechMed-Graz, Petersgasse 12/2, A-8010 Graz, Austria; 3 Institute of Biophysics, Medical University of Graz, BioTechMed-Graz, Harrachgasse 21, A-8010 Graz, Austria; 4 Department of Biological Sciences, University of Alberta, CW405 Biological Sciences Building, Edmonton, Alberta, Canada T6G 2E9

## Abstract

Bacterial outer membrane vesicles (OMVs) have important biological roles in pathogenesis and intercellular interactions, but a general mechanism of OMV formation is lacking. Here we show that the VacJ/Yrb ABC (ATP-binding cassette) transport system, a proposed phospholipid transporter, is involved in OMV formation. Deletion or repression of VacJ/Yrb increases OMV production in two distantly related Gram-negative bacteria, *Haemophilus influenzae* and *Vibrio cholerae*. Lipidome analyses demonstrate that OMVs from VacJ/Yrb-defective mutants in *H. influenzae* are enriched in phospholipids and certain fatty acids. Furthermore, we demonstrate that OMV production and regulation of the VacJ/Yrb ABC transport system respond to iron starvation. Our results suggest a new general mechanism of OMV biogenesis based on phospholipid accumulation in the outer leaflet of the outer membrane. This mechanism is highly conserved among Gram-negative bacteria, provides a means for regulation, can account for OMV formation under all growth conditions, and might have important pathophysiological roles *in vivo*.

## Abstract (web summary)

Bacteria release outer membrane vesicles (OMVs) that play important roles in pathogenesis and intercellular interactions. Here, Roier *et al*. provide evidence supporting that phospholipid accumulation in the outer leaflet of the outer membrane participates in OMV formation in Gram-negative bacteria.

## Graphical abstract

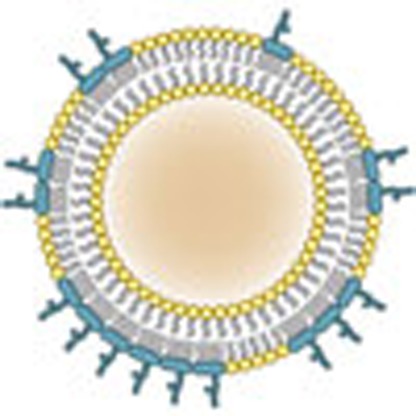

Outer membrane vesicles (OMVs) are spherical, bilayered, membranous structures that are released naturally from the outer membrane (OM) of Gram-negative bacteria^1^. These small particles (∼10–300 nm in diameter) are primarily composed of phospholipids (PLs), OM proteins (OMPs), and lipopolysaccharides or lipooligosaccharides, but also contain periplasmic proteins and cell wall components, which get trapped in the vesicle lumen during the release process. However, OMVs may also comprise proteins of the inner membrane, cytoplasmic proteins, DNA, RNA, ions, metabolites and signalling molecules^2^,^3^,^4^. The secretion of OMVs seems to be an evolutionary conserved process, as OMV production has been observed under various growth conditions and in different natural environments in all pathogenic and non-pathogenic Gram-negative bacteria investigated so far. Since OMV production requires a significant energy cost, the benefits in certain environmental conditions must have been sufficient to allow OMV secretion to evolve^1^,^2^,^5^,^6^. Based on their metabolic cost, ubiquity and interesting cargo material, it is likely that OMVs have vital biological functions and, as a consequence of intense research, it was soon found that OMVs play important roles in bacterial physiology and pathogenesis. They are proposed to be involved in horizontal gene transfer, biofilm formation, intra- and interspecies communication, stress response, delivery of toxins and other biomolecules, killing of competing microbial cells, resistance to antibiotics, adherence to host cells, complement absorption and immunomodulation^1^,^2^,^3^,^5^,^7^. In addition, many studies focused on vesicle-based vaccines and demonstrated that OMVs are promising vaccine candidates against bacterial infections caused by, for example, *Haemophilus influenzae*, *Pasteurella multocida*, *Vibrio cholerae*, enterotoxigenic *Escherichia coli*, *Neisseria meningitidis*, *Bordetella pertussis* and *Salmonella typhimurium*^8^,^9^,^10^,^11^,^12^,^13^.

Despite these important biological roles and applications of OMVs, research has only just begun to understand the mechanism of OMV biogenesis. Currently, three major models are discussed within the scientific community. The first model is based on either loss or relocation of covalent linkages (for example, via lipoproteins) between the OM and the underlying peptidoglycan layer. These missing cross-links in combination with an OM that grows faster than the underlying cell wall allow the OM to protrude and thus initiate vesiculation^2^,^3^,^7^,^14^,^15^,^16^. A second model proposes that an accumulation of peptidoglycan fragments or misfolded proteins in the periplasmic space exerts a turgor pressure on the OM causing the OM to bulge and finally to pinch off. Such accumulations can be triggered by, for example, defects in cell wall remodelling or temperature stress^2^,^3^,^7^,^17^,^18^. The third model is based on the enrichment of membrane curvature-inducing molecules, such as the B-band lipopolysaccharide and the quinolone PQS of *Pseudomonas aeruginosa*. It is hypothesized that PQS enhances anionic repulsions between lipopolysaccharide molecules resulting in membrane blebbing by sequestering divalent cations, which are important in forming stabilizing salt bridges between the negatively charged B-band lipopolysaccharide molecules. Recently, it was proposed that PQS induces OMV formation through a mechanism of asymmetric expansion of the outer leaflet of the OM^2^,^3^,^7^,^19^,^20^,^21^,^22^. Although the PQS-based model is one of the best studied so far, it is limited by the fact that PQS is only produced by *P. aeruginosa* and therefore species specific. In summary, all these proposed models of OMV formation require either genetic manipulations, the presence of stress, or are thus far only applicable to a single bacterial species. It is currently unknown whether Gram-negative bacteria share a conserved general mechanism of OMV biogenesis that is amenable to regulation.

Here we show that disruptions within the VacJ/Yrb ABC (ATP-binding cassette) transport system increase OMV production in the human pathogens *H. influenzae* and *V. cholerae* without compromising OM integrity. Concordantly, mutations in homologues of *E. coli* also exhibit increased vesiculation, as independently demonstrated by other groups (ref. 23 and Thomas J. Silhavy, Princeton University, personal communication). Since this system is proposed to function as a PL transporter in maintaining the lipid asymmetry in the OM^24^, we performed lipidome analyses to demonstrate that OMVs from PL transporter mutants in *H. influenzae* are enriched in PLs, which are likely to be incorporated into the outer leaflet of the vesicle membrane. Furthermore, we report that iron limitation leads to a ferric uptake regulator (Fur)-dependent downregulation of the VacJ/Yrb ABC transport system correlating with an increased OMV production in *H. influenzae*, *V. cholerae* and *E. coli*. Our results indicate that the disadvantage of an increased serum sensitivity caused by this downregulation can be overcome *in vivo* by an increased OMV production suggesting an important pathophysiological role of this system. We propose a novel and potentially highly conserved bacterial OMV biogenesis mechanism, which provides the opportunity of regulation and may represent a first general mechanism applicable to all Gram-negative bacteria.

## Results

### Identification of mutants with altered OMV production

**Figure 1:**
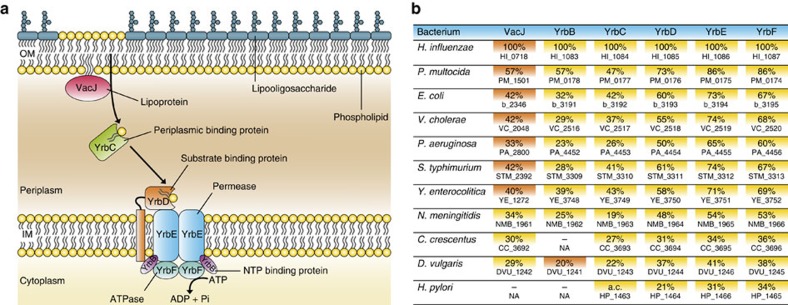
A conserved ABC transport system putatively prevents PL accumulation in the outer leaflet of the OM. (**a**) The VacJ and Yrb proteins of *H. influenzae* are homologous to members of the *E. coli* Mla pathway, which has been proposed to maintain the lipid asymmetry in the Gram-negative OM by retrograde trafficking of PLs from the OM to the inner membrane (IM)^24^. The model is derived from the Mla pathway model proposed by Malinverni and Silhavy^24^. (**b**) Conservation of *H. influenzae* VacJ and Yrb proteins among Gram-negative bacteria. The respective genes or gene clusters were identified via the SSEARCH programme in combination with the SSDB (Similarity Sequence DataBase) using the KEGG database resource. Shown are the corresponding gene numbers and the respective amino acid sequence identities (in percentage), which were determined by BLASTp and/or Clustal Omega. Adjacent genes located in gene clusters are highlighted in yellow, genes located outside of the *yrb* gene clusters are coloured orange. In case of a single dash and NA (not applicable) no conserved homologous protein was identified. In *Helicobacter pylori* an auxiliary component (a.c.) with no homology to any other component is encoded within the *yrb* gene cluster. Genomes analysed were: *H. influenzae* Rd KW20 (γ), *P. multocida* Pm70 (γ), *E. coli* K-12 MG1655 (γ), *V. cholerae* O1 El Tor N16961 (γ), *P. aeruginosa* PAO1 (γ), *Salmonella enterica* serovar Typhimurium LT2 (γ), *Yersinia enterocolitica* 8081 (γ), *N. meningitidis* MC58 (β), *Caulobacter crescentus* CB15 (α), *Desulfovibrio vulgaris* Hildenborough (δ) and *H. pylori* 26695 (ɛ). The symbols α, β, γ, δ and ɛ refer to the class of the respective bacterium within the phylum *Proteobacteria*.

In an attempt to discover a general mechanism of OMV formation, we used *H. influenzae*, an opportunistic pathogen of the human respiratory tract^25^, as a model organism. We conducted a transposon mutagenesis and a dot blot screen to identify 20 gene disruptions that led to altered OMV production based on immunological detection of several OMV associated proteins (Supplementary Table 1). For this analysis OMPs P1, P2, P4, P5 and P6 were chosen, since these represent highly abundant OMPs of *H. influenzae* and have recently shown to be present in OMVs in detectable levels^26^. Representative examples for dot blots as well as examples for the evaluation of relative signal intensities are provided in Supplementary Fig. 1. In most cases the observed trends generally correlated with an increase or decrease of the surveyed OMPs P1, P2, P4, P5 or P6 in OMVs indicating no differential protein sorting. Notably, the majority of transposon insertion mutants showed markedly and consistently increased relative OMV and OMP dot blot signals suggesting an overall increase in vesiculation, while only four mutants exhibited decreased dot blot signals indicating hypovesiculation. A similar trend has also been reported for *E. coli* in a previous screen for alterations in OMV production^16^. The most pronounced phenotypes for hypovesiculation were observed for insertion mutants in HI_0572 and HI_0854 encoding for a peroxiredoxin hybrid Prx5 and a haem iron utilization protein, respectively. Strong hypervesiculation phenotypes were observed for insertion mutants in HI_0037 (rod shape-determining protein MreB), HI_0528 (tyrosine-specific transport protein TyrP), HI_1083 (NTP-binding protein YrbB), HI_1086 (ABC transporter permease YrbE), HI_1164 (OMP P5), HI_1181 (phosphoheptose isomerase GmhA) and HI_1213 (thiol-disulfide interchange protein DsbC). An increased vesiculation due to deletion or truncation of OmpA, representing the OMP P5 homologue and an abundant protein linking the OM and peptidoglycan layer, has already been reported in *E. coli*, *Salmonella*, and *V. cholerae*^15^,^27^,^28^. Other mutants, including those in *mreB*, *dsbC* and *gmhA* can be linked to OM stability or stress response, which have been previously implicated in OMV secretion^2^,^7^,^15^,^18^. In this study we focused on adjacent mutations in HI_1083 (*yrbB*) and HI_1086 (*yrbE*), which showed strong OMV and OMP dot blot signals suggesting an overall increase in vesiculation. These were the most promising candidates for further evaluation, as they have not been previously implicated in OMV formation and both genes are located within a gene cluster comprising three additional genes (*yrbC*, *yrbD* and *yrbF*). In *E. coli*, homologues of these five gene products, together with the VacJ protein, have been implicated in maintaining the lipid asymmetry in the Gram-negative OM^24^. This ABC transport system was termed the Mla pathway and is thought to prevent PL accumulation in the outer leaflet of the OM by retrograde trafficking of PLs from the OM to the inner membrane^24^. Recent studies reported that this PL transport system also contributes to serum resistance among pulmonary isolates of nontypeable *H. influenzae* (NTHi)^29^ and is required for intercellular spread of *Shigella flexneri*^30^. Figure 1a illustrates the putative VacJ/Yrb ABC transport system in *H. influenzae* based on the *E. coli* Mla pathway model^24^. Notably, the VacJ and Yrb proteins of *H. influenzae* as well as the overall genomic organization of their corresponding genes are conserved among Gram-negative bacteria (Fig. 1b). In representatives of β-, γ- and δ-*Proteobacteria* all components are highly conserved, while α- and ɛ-*Proteobacteria* are lacking the smallest component annotated as NTP-binding protein YrbB. In addition, ɛ-*Proteobacteria* have an auxiliary periplasmic substrate-binding component instead of the periplasmic binding protein YrbC and the OM lipoprotein VacJ. The integral components of the transport system including the substrate-binding protein YrbD, the inner membrane permease YrbE and the ATPase YrbF are highly conserved in all representatives. Indeed, the conservation of core components of the VacJ/Yrb ABC transport system in Gram-negative bacteria and in the chloroplasts of plants has also been previously reported by others^24^,^31^.

### VacJ/Yrb ABC transport system mutants produce more OMVs

**Figure 2:**
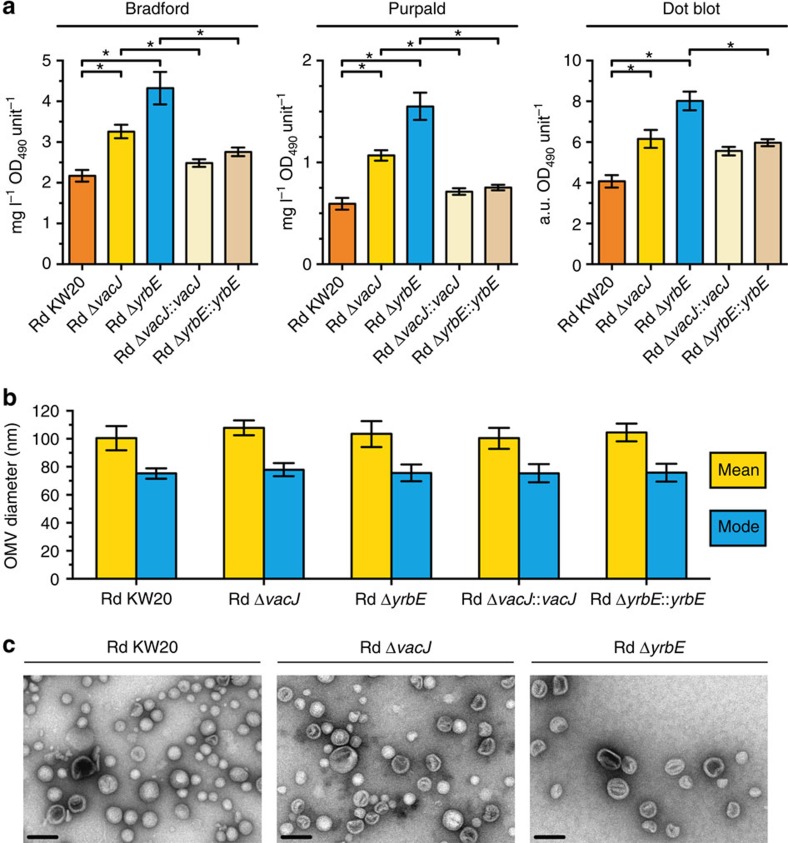
***H. influenzae*** **PL transporter mutants produce excessive OMVs.** (**a**) OMV preparations derived from equivalent OD_490_ units of cultured wild-type (Rd KW20), deletion mutants (Rd Δ*vacJ* and Rd Δ*yrbE*) and complementation strains (Rd Δ*vacJ*::*vacJ* and Rd Δ*yrbE*::*yrbE*) were analysed for total protein (Bradford), lipooligosaccharide (Purpald) or OMV specific proteins (Dot blot). Mean values with standard error of the mean (s.e.m.) are shown (*n*=6 biological replicates). Significant differences between the data sets are marked by asterisks (*P*<0.05; one-way ANOVA followed by Sidak's multiple comparison post test). (**b**) Distributions of OMV sizes produced by the strains were determined by nanoparticle tracking analysis. Mean values with standard deviation (s.d.) of mean and mode OMV diameter sizes within each OMV preparation are shown (*n*=6 biological replicates). (**c**) Visualization of density gradient purified OMVs by transmission electron microscopy. Shown are representative micrographs. Scale bars, 100 nm.

**Figure 3:**
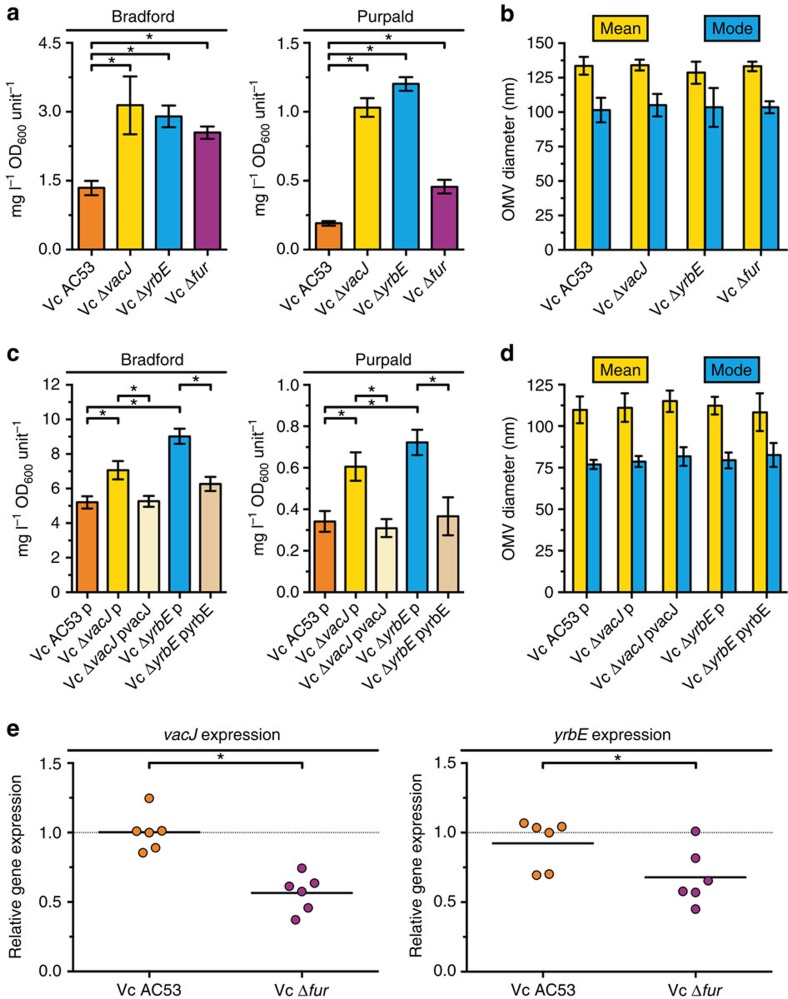
Excessive OMV production by PL transporter mutants, and regulation of *vacJ* and *yrbE* expression in *V. cholerae*. (**a**,**c**) OMV preparations derived from equivalent OD_600_ units of cultured wild-type (Vc AC53) and deletion mutants (Vc Δ*vacJ*, Vc Δ*yrbE* and Vc Δ*fur*) as well as wild-type containing vector control (Vc Ac53 p) and deletion mutants containing either vector control (Vc Δ*vacJ* p, Vc Δ*yrbE* p) or expression plasmids (Vc Δ*vacJ* pvacJ and Vc Δ*yrbE* pyrbE) were analysed for total protein (Bradford) and lipopolysaccharide (Purpald). Mean values with s.e.m. are shown (*n*=12 biological replicates for Vc AC53 and *n*=6 biological replicates for all other strains). (**b**,**d**) Distributions of OMV sizes produced by the strains were determined by nanoparticle tracking analysis. Mean values with s.d. of mean and mode OMV diameter sizes within respective OMV preparations are shown (*n*=12 biological replicates for Vc AC53 and *n*=6 biological replicates for all other strains). (**e**) Relative expressions of *vacJ* and *yrbE* in Vc AC53 and Vc Δ*fur* were determined by qRT-PCR. Horizontal bars highlight the mean of each data set (*n*=6 biological replicates). Dotted lines indicate a relative gene expression of 1. (**a**,**c**,**e**) Significant differences between the data sets are marked by asterisks (*P*<0.05; one-way ANOVA followed by Sidak's multiple comparison post test (**a**,**c**) or unpaired t test (**e**)).

To confirm that disruptions within the VacJ/Yrb ABC transport system increase OMV production in *H. influenzae*, we constructed gene-specific *vacJ* and *yrbE* deletion mutants and quantified the protein and lipooligosaccharide content of their derived OMVs using established methodologies^16^,^26^ (Fig. 2a). Compared with the wild-type, deletions of the OM lipoprotein VacJ or the inner membrane permease YrbE revealed significant increases in OMV production with, on average, 1.6-fold and 2.2-fold higher vesiculation levels, respectively. The hypervesiculation phenotypes of nonpolar deletion mutants were complementable. To ensure that the higher amounts of protein and lipooligosaccharide as well as the elevated dot blot signals detected in OMV preparations were really due to increased vesiculation levels and not due to increased vesicle sizes, we performed a nanoparticle tracking analysis (Fig. 2b) and confirmed these results by transmission electron microscopy (Fig. 2c). We found no differences in the OMV size distribution between the strains confirming that the deletion of *vacJ* or *yrbE* indeed increases the amount of OMVs produced by *H. influenzae*. Sensitivity of the mutant strains to cell lysis, SDS and polymyxin B treatment was comparable to the wild-type indicating that the OM integrity remains largely intact (Supplementary Fig. 2). Similar observations were also reported for related genes^24^,^29^, strengthening the conclusion that the hypervesiculation phenotypes are not caused by a compromised OM integrity. To determine whether these findings also hold true in a distantly related Gram-negative species, we constructed *vacJ* and *yrbE* deletion mutants in *V. cholerae* and determined their vesiculation levels and OMV size distributions (Fig. 3a,b). Hypervesiculation phenotypes similar to those of *H. influenzae* were observed. PL transporter mutants in *V. cholerae* revealed, on average, 3.9-fold (Δ*vacJ*) and 4.3-fold (Δ*yrbE*) higher vesiculation levels but similar OMV sizes compared with the wild-type. Similar to the complementation analysis in *H. influenzae*, the hypervesiculation phenotypes of the Δ*vacJ* and Δ*yrbE* mutants in *V. cholerae* were restored to wild-type levels by expression of the respective gene in trans (Fig. 3c,d). Concordantly, mutations in the *E. coli* Mla pathway exhibit increased vesiculation, as independently demonstrated by another group (Thomas J. Silhavy, Princeton University, personal communication). This observation is reinforced by a recent genome wide assessment of OMV production in *E. coli* indicating an increased vesiculation of *mlaA* (*vacJ*) as well as *mlaE* (*yrbE*) mutants, which was not further investigated^23^. Furthermore, we used the arabinose-inducible vector system pBAD to overexpress the *mla* gene cluster (comprising *mlaF* to *B*) in the *E. coli* wild-type. Consistent, with the increase in vesiculation upon deletion of the Mla pathway, the overexpression resulted in decreased vesiculation compared with the empty vector control (Supplementary Fig. 3). Taken together, these findings indicate a conserved role of the VacJ/Yrb ABC transport system in OMV formation of distantly related Gram-negative bacteria.

### OMVs from PL transporter mutants have an altered lipidome

To evaluate the OMV and OM compositions of the *H. influenzae* wild-type and PL transporter mutants Δ*vacJ* and Δ*yrbE*, we performed proteome and lipidome analyses. Comparison of protein profiles generated by SDS–PAGE (Supplementary Fig. 4) and proteome analysis by mass spectrometry (Supplementary Table 2) revealed very similar OMV or OM protein compositions for each strain, in good agreement with the dot blot results (Supplementary Table 1). Although the PL transporter mutants showed a slight alteration in the high molecular weight OMV protein band pattern in the SDS–PAGE, we did not follow this observation, since it was not restored by complementation (Supplementary Fig. 4). Additionally, the protein mass spectrometry results (Supplementary Table 2) of the OMVs revealed no relevant hits in the PL transporter mutant OMVs compared with the wild-type OMVs, which could explain this alteration in protein band patterns. Therefore, we conclude that the PL transporter mutants closely resemble the wild-type regarding OMV or OM protein compositions, respectively.

**Figure 4:**
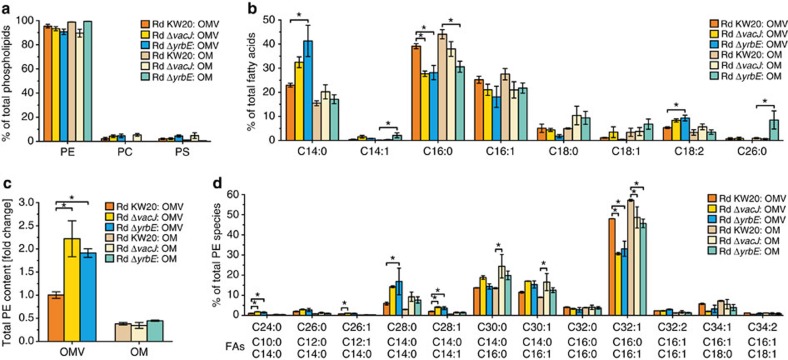
Lipidome analyses of *H. influenzae* density gradient purified OMVs and OM. (**a**,**b**) PL (**a**) and FA (**b**) compositions of OMV and OM preparations derived from Rd KW20, Rd Δ*vacJ*, and Rd Δ*yrbE* were analysed by thin-layer chromatography and by gas liquid chromatography in combination with flame-ionization detection, respectively. Mean percentage values with s.e.m. of total PLs (**a**) and FAs (**b**) within a given preparation are shown (*n*=3 biological replicates). Detected PLs and FAs were: phosphatidylethanolamine (PE), phosphatidylcholine (PC), phosphatidylserine (PS), myristic acid (C14:0), myristoleic acid (C14:1), palmitic acid (C16:0), palmitoleic acid (C16:1), stearic acid (C18:0), oleic acid (C18:1), linoleic acid (C18:2), and cerotic acid (C26:0). (**c**,**d**) Total PE contents (**c**) and PE species compositions (**d**) of respective OMV and OM preparations were determined by LC/ESI-MS. Total PE contents (**c**) are given in *x*-fold changes normalized to Rd KW20 OMV preparations and PE species compositions (**d**) are given in percentage of total PE species within a respective preparation. Mean values with s.e.m. are shown (*n*=3 biological replicates). For PE composition analysis (**d**), only PE species over 1% (at least in one preparation) are shown. The main FAs of a given PE species are indicated below each species. Additional detected FAs: capric acid (C10:0), lauric acid (C12:0), and lauroleic acid (C12:1). (**b**,**c**,**d**) Significant differences between the data sets are marked by asterisks (*P*<0.05; one-way ANOVA followed by Sidak's multiple comparison post test).

Analysis of the PL composition confirmed the results of our recent study^26^, which demonstrated that phosphatidylethanolamine (PE) is by far the most dominant PL species identified in OMVs and the OM of *H. influenzae* (Fig. 4a). Only minor alterations in phosphatidylcholine and phosphatidylserine levels in OM preparations of the strains were observed, which did not correlate with increased or decreased vesiculation and were therefore not further investigated. Other differences in OMV or OM PL compositions between the strains were negligible. In contrast, analysis of fatty acid (FA) composition revealed that the OMVs of PL transporter mutants show significant 10 percentage point decreases in C16:0 FA levels compared with the wild-type, which generally goes along with significant 8 to 15 percentage point increases of C14:0 FA levels (Fig. 4b). By using a complementary mass spectrometry-based lipidome analysis and by analysing identical amounts (protein equivalents) of respective preparations, we were able to compare quantitatively the total PE contents of all OMV and OM preparations (Fig. 4c). Total OM PE contents were not altered among the strains, whereas the total PE contents of OMVs derived from the PL transporter mutants were significantly twofold increased compared with wild-type OMVs. Similar trends of slightly increased PL levels were already visible in the wild-type OMVs compared with the wild-type OM, but became more pronounced in the mutant strains (Fig. 4c). In consideration of the similar OMV size distributions, these results indicate that OMVs from PL transporter mutants are enriched in PLs, which are likely to be incorporated into the outer leaflet of the vesicle membrane.

To exclude the possibility that the observed alterations in PL levels result from increased expression of FA biosynthesis genes in the PL transporter mutants, we performed qRT-PCR analyses of the genes *fabB*, *fabD* and *fabH*. These genes encode for the 3-oxoacyl-ACP synthase I, the acyl carrier protein S-malonyltransferase, and the 3-oxoacyl-ACP synthase III representing three key enzymes in the FA biosynthesis pathway. The *H. influenzae* Δ*yrbE* mutant was chosen for direct comparison to the wild-type, since it showed the most pronounced alterations in the lipidome. As Δ*yrbE* and the wild-type exhibited similar transcriptional levels for all three genes (Supplementary Fig. 5), the observed PL accumulation in PL transporter mutants is not due to increased FA biosynthesis.

PE species composition analysis (Fig. 4d) confirmed the results of the FA analysis (Fig. 4b) by showing reduced PE species containing C16:0 and enriched PE species containing C14:0 in OMVs derived from PL transporter mutants compared with the wild-type. Notably, the C16:0/C14:0 FA shift (Fig. 4b,d) as well as the difference in total PE contents (Fig. 4c) between OMVs and the OM were already present in the wild-type and were just more pronounced in the PL transporter mutants. Since these changes in the PL content correlate with increased OMV formation, we hypothesize that defined PL rearrangements promote OMV formation in *H. influenzae*.

### Fur regulates expression of the PL transporter

**Figure 5:**
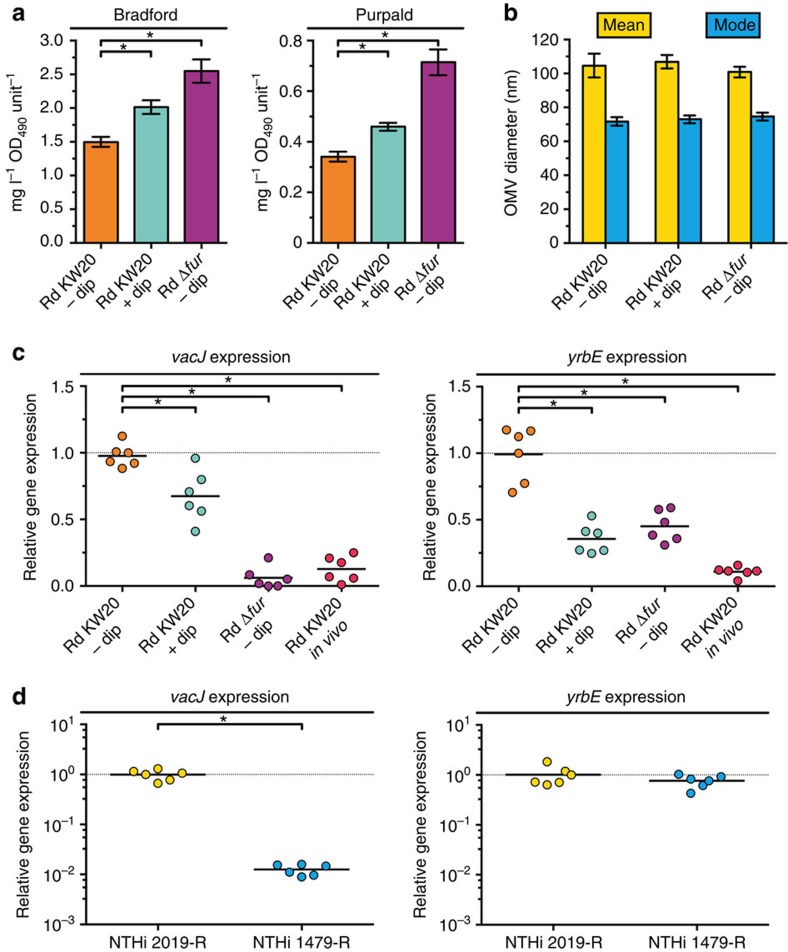
Regulation of *vacJ* and *yrbE* expression in *H. influenzae*. (**a**) OMV preparations derived from equivalent OD_490_ units of cultured Rd KW20 or Rd Δ*fur* grown in BHI–NAD–hemin supplemented without (−dip) or with (+ dip) 2,2′-dipyridyl were analysed for total protein (Bradford) and lipooligosaccharide (Purpald). Mean values with s.e.m. are shown (*n*=6 biological replicates). (**b**) Distributions of OMV sizes produced by the strains in different culture conditions were determined by nanoparticle tracking analysis. Mean values with s.d. of mean and mode OMV diameter sizes within respective OMV preparations are shown (*n*=6 biological replicates). (**c**) Relative expressions of *vacJ* and *yrbE* in Rd KW20 or Rd Δ*fur* grown in BHI–NAD–hemin supplemented without (−dip) or with (+ dip) 2,2′-dipyridyl as well as in Rd KW20 after colonization of the mouse nasopharynx for 4 h (*in vivo*) were determined by qRT-PCR. (**d**) Relative expressions of *vacJ* and *yrbE* in NTHi 2019-R and NTHi 1479-R grown in BHI–NAD–hemin were determined by qRT-PCR. (**c**,**d**) Horizontal bars highlight the mean of each data set (*n*=6 biological replicates). Dotted lines indicate a relative gene expression of 1. (**a**,**c**,**d**) Significant differences between the data sets are marked by asterisks (*P*<0.05; one-way ANOVA followed by Sidak's multiple comparison post test (**a,c**) or unpaired *t*-test (**d**)).

**Figure 6:**
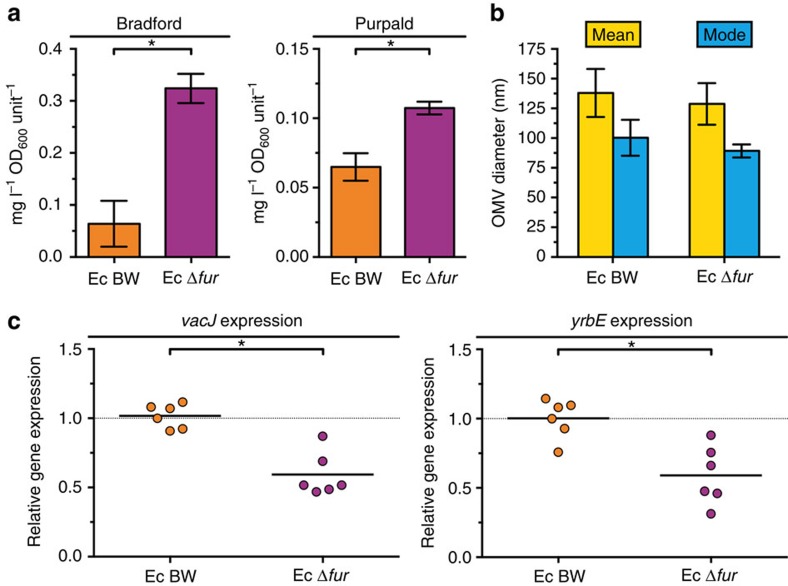
Regulation of *vacJ* and *yrbE* expression in *E. coli*. (**a**) OMV preparations derived from equivalent OD_600_ units of cultured wild-type (Ec BW) and deletion mutant (Ec Δ*fur*) were analysed for total protein (Bradford) and lipooligosaccharide (Purpald). Mean values with s.e.m. are shown (*n*=6 biological replicates). (**b**) Distributions of OMV sizes produced by the strains were determined by nanoparticle tracking analysis. Mean values with s.d. of mean and mode OMV diameter sizes within respective OMV preparations are shown (*n*=6 biological replicates). (**c**) Relative expressions of *vacJ* and *yrbE* in Ec BW and Ec Δ*fur* were determined by qRT-PCR. Horizontal bars highlight the mean of each data set (*n*=6 biological replicates). Dotted lines indicate a relative gene expression of 1. (**a**,**c**) Significant differences between the data sets are marked by asterisks (*P*<0.05; unpaired *t*-test).

By comparing the OMV quantification results of our *H. influenzae* wild-type strain Rd KW20 with those of our previous study^26^, it became obvious that cultures containing protoporphyrin IX instead of hemin solution produce more OMVs. Besides presence of L-histidine and triethanolamine in the hemin solution, the only other difference between cultures containing protoporphyrin IX and hemin solution is the presence of iron contained in hemin^32^. Thus, we considered whether iron availability might be involved in the regulation of OMV formation in *H. influenzae*. To address this question, we first determined the vesiculation levels and OMV size distributions of Rd KW20 grown in hemin medium supplemented with or without the iron chelator 2,2′-dipyridyl^33^ (Fig. 5a,b). We found that Rd KW20 produces 35% more, but similarly sized, OMVs in the presence of 2,2′-dipyridyl. qRT-PCR experiments revealed that *vacJ* and *yrbE* expression levels were significantly decreased (∼two-fold) in Rd KW20 grown under iron-restricted conditions mediated by addition of 2,2′-dipyridyl (Fig. 5c). This indicates that iron limitation leads to a downregulation of the VacJ/Yrb ABC transport system, which ultimately results in increased OMV production in *H. influenzae*. Furthermore, we asked whether OMV production and the expression of the PL transporter genes is influenced by Fur, a global transcriptional repressor or activator of iron-regulated genes in most bacteria^34^,^35^,^36^. Indeed, a Δ*fur* mutant grown under iron-replete conditions produces at least 70% more, but similarly sized OMVs (Fig. 5a,b). Additional qRT-PCR experiments performed with the Δ*fur* mutant revealed that *vacJ* and *yrbE* expression levels were significantly decreased (∼10-fold and twofold, respectively) compared with Rd KW20 grown under the same conditions (Fig. 5c). This suggests that Fur activates *vacJ* and *yrbE* expression and that the downregulation of the PL transporter genes in a Δ*fur* mutant correlates with increased OMV production in *H. influenzae*. To investigate whether this also holds true in distantly related Gram-negative bacteria, we analysed the OMV production and expression levels of PL transporter genes in Δ*fur* mutants of *V. cholerae* and *E. coli*. In accordance with the observations in *H. influenzae* (Fig. 5), Δ*fur* mutants of *V. cholerae* and *E. coli* exhibited a significant increase in vesiculation of about twofold and a significant twofold decrease in *vacJ* and *yrbE* transcription compared with the respective wild-type (Figs 3a,b,e; 6). Thus, hypervesiculation phenotypes of Δ*fur* mutants correlate with downregulation of *vacJ* and *yrbE* in all three bacterial species. Furthermore, *in silico* analyses revealed putative Fur binding sites in the upstream regions of *vacJ* and the *yrb* gene clusters of *H. influenzae*, *V. cholerae* and *E. coli*, respectively (Supplementary Fig. 6). Although this provides a first hint that Fur might interact directly with the respective upstream regions, we cannot exclude that the Fur-dependent activation observed in this study may also be indirect via a yet unknown factor. In summary, these results indicate that the observed increase in vesiculation correlates with a downregulation of the PL transporter genes via a Fur-dependent manner, which is conserved in distantly related Gram-negative bacteria.

As iron limitation is commonly observed for bacterial pathogens in the host^37^,^38^, we analysed the expression levels of the PL transporter genes in *H. influenzae* Rd KW20 during the initial stages of nasopharyngeal colonization. Compared with the *in vitro* condition using BHI–NAD–hemin as a growth medium, Rd KW20 revealed significantly and at least sevenfold decreased *vacJ* and *yrbE* expression levels after colonization of the mouse nasopharynx for 4 h (Fig. 5c). The reduced PL transporter gene expression *in vivo* suggests an increased OMV production by bacterial pathogens during initial colonization of the host. Furthermore, we recently observed different levels of OMV production for clinical NTHi isolate 2019-R and 1479-R, with the latter generating about twofold more OMVs^26^. In the light of the data presented herein, we investigated the PL transporter gene expressions in these two clinical NTHi isolates and observed that *vacJ* expression levels were significantly 100-fold decreased in NTHi 1479-R compared with those in NTHi 2019-R (Fig. 5d), which is consistent with the observation that NTHi 1479-R generates more OMVs compared with NTHi 2019-R (ref. 26).

### Hypervesiculation correlates with serum resistance

**Figure 7:**
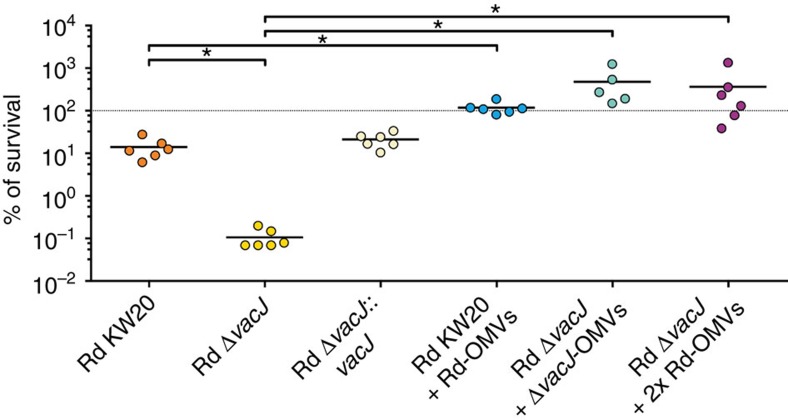
OMVs increase the serum resistance of *H. influenzae*. Serum bactericidal assays were used to determine the serum resistance of Rd KW20, Rd Δ*vacJ*, and Rd Δ*vacJ*::*vacJ* to normal human serum. Groups tagged with ‘+ Rd-OMVs', ‘+ Δ*vacJ*-OMVs', and ‘+ 2x Rd-OMVs' indicate assays, where Rd KW20 or Rd Δ*vacJ* OMV preparations were added to corresponding assays using respective physiological final *in vitro* concentrations or a twofold higher concentration (2 × Rd-OMVs). For details please see the respective method section (serum bactericidal assay). Horizontal bars highlight the mean percentage of survival of each group (*n*=5–6 biological replicates). The dotted line indicates 100% survival. Significant differences between the data sets are marked by asterisks (*P*<0.05; one-way ANOVA followed by Sidak's multiple comparison post test).

Interestingly, a recent study revealed that many clinical isolates of NTHi have surprisingly low *vacJ* and *yrbE* expression levels and these correlate with increased serum sensitivity^29^. Consistent with this previous report for NTHi, the *H. influenzae* Rd Δ*vacJ* mutant showed a reduced survival rate in presence of human serum compared with the wild-type Rd KW20, which was restored by complementation (Fig. 7). Here we demonstrated that serum resistance was also significantly 4,000-fold increased to a Δ*vacJ* mutant *in vitro* by the addition of physiological concentrations of OMVs derived from a Δ*vacJ* mutant. Likewise, the wild-type showed a significant 10-fold increase in serum resistance, when physiological concentrations of OMVs derived from the wild-type were added. To exclude differences in the capacity to resist complement-mediated attacks, we also performed an assay using twofold higher wild-type OMV concentrations added to the Δ*vacJ* mutant. As the Δ*vacJ* mutant produces approximately twice as many OMVs compared with the wild-type (Fig. 2), the double amount of wild-type OMVs reflect a physiological concentration of Δ*vacJ* OMVs. Since the survival rate of the Δ*vacJ* mutant in presence of Δ*vacJ* OMVs and in presence of the same amount of wild-type OMVs is quite similar, we can exclude a differential capacity between wild-type and mutant OMVs to resist complement activation. These data suggest that the disadvantage of an increased serum sensitivity caused by a downregulation of the VacJ/Yrb ABC transport system can be overcome *in vivo* by an increased OMV production.

## Discussion

**Figure 8:**
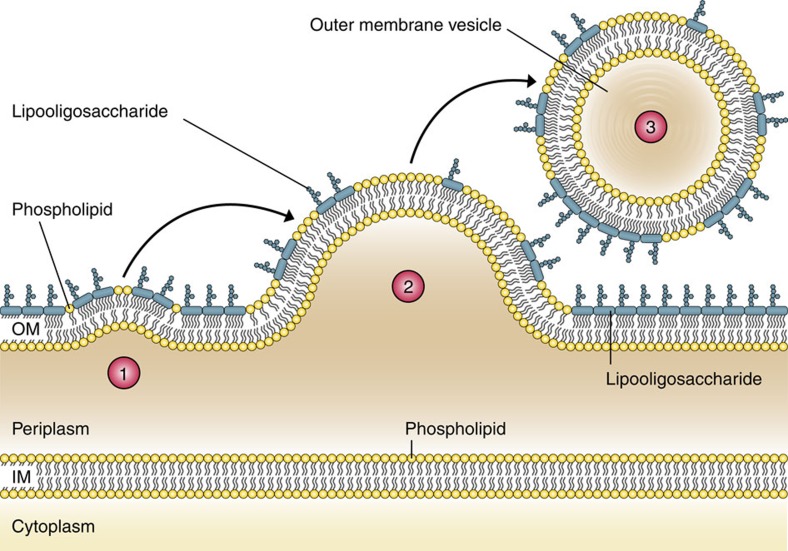
A new model of OMV formation in Gram-negative bacteria. Step 1: Decreased expression or deletion of *vacJ* and/or *yrb* genes results in PL accumulation in the outer leaflet of the OM. This asymmetric expansion of the outer leaflet initiates an outward bulging of the OM. Step 2: Further enrichment of positive and negative curvature-inducing PLs in both leaflets supports the budding of the OM, which finally pinches off to form an OMV. Step 3: The released OMV is enriched in PLs incorporated into the outer leaflet of the vesicle membrane.

Taken together, our findings allow us to propose a novel and potentially highly conserved OMV biogenesis mechanism in Gram-negative bacteria (Fig. 8). According to this model, deletion of *vacJ* and/or *yrb* genes, or their reduced expression, results in PL accumulation in the OM. As Δ*vacJ* and Δ*yrbE* mutants do not exhibit higher PL contents in their OM compared with the wild-type, accumulated PLs are likely to be directly secreted via OMVs. Concordantly, OMVs derived from the PL transporter mutants contain higher PL levels compared with wild-type OMVs. Notably, all hypervesiculating strains as well as the wild-type investigated in this study secrete OMVs of comparable size distribution, indicating a similar amount of PLs in the inner leaflet of the vesicle membrane. Thus, the observed enrichment of PLs in OMVs derived from PL transporter mutants implicates a PL incorporation in the outer leaflet of the vesicle membrane. Since the PL transporter genes are highly conserved among Gram-negative bacteria and the hypervesiculation phenotype of PL transporter mutants can be observed in distantly related Gram-negative bacteria (for example, *H. influenzae*, *V. cholerae* and *E. coli*), this model of OMV formation could be a general mechanism applicable to a variety of Gram-negative bacteria.

Moreover, we show that this mechanism can be regulated by iron availability in a Fur-dependent manner. Although additional regulatory pathways may act on the PL transporter genes, it is likely that iron limitation causes the observed downregulation of the respective genes *in vivo*, which might impact the bacterial pathophysiology. One example could be the transmission of *H. influenzae* into a new host. Upon initial colonization of the nasopharynx, *H. influenzae* must overcome mucosal immune defence mechanisms, including the protective effects of complement factors and secretory IgA antibodies^39^,^40^. Based on our findings, it can be hypothesized that the iron-limiting conditions *in vivo* cause a downregulation of the VacJ/Yrb ABC transport system resulting in increased OMV production, which counteracts antibody and complement attacks. The importance of OMVs in bacterial serum resistance is also highlighted by a recent study by Tan *et al*.^41^ demonstrating that *Moraxella catarrhalis* OMVs are involved in the complement resistance of nasopharyngeal bacteria by binding and depleting complement factors. One could speculate that this might also be a protective feature of *H. influenzae* OMVs within the human nasopharynx. The hypervesiculation during initial colonization of a new host could provide an advantage for *H. influenzae* by lowering the selective pressure of local immune defence mechanisms and thereby facilitating proliferation in the nasopharynx. In this sense, our proposed OMV biogenesis mechanism could be very important for bacterial adaptation to a new host.

It should be emphasized that this OMV biogenesis mechanism based on PL accumulation can act in concert with all other OMV formation models proposed so far. As already briefly discussed in the introduction, previously reported OMV formation models are species-specific, require the presence of stress or depend on mutations affecting OM integrity. In contrast, the model proposed herein overcomes these limitations as it represents a first general mechanism that can account for OMV formation under all growth conditions, is applicable for a broad range of Gram-negative bacteria, and can be regulated by the microorganisms.

## Methods

### Ethics statement

Female BALB/c (Charles River Laboratories) were used for all immunization experiments and female CD-1 IGS mice (Charles River Laboratories) were used for colonization studies in strict accordance with the recommendations in the Guide for the Care and Use of Laboratory Animals of the National Institutes of Health, the national ‘Bundesgesetzblatt für die Republik Österreich'. The corresponding animal protocol (39/53/00 ex 2012/13) has been approved by the Austrian Federal Ministry of Science and Research Ref. II/10b and the local Committee on the Ethics of Animal Experiments of the University of Graz. Mice were housed with food and water *ad libitum* and monitored under the care of full-time staff and in accordance with the rules of the Institute of Molecular Biosciences at the University of Graz. All animals were acclimated for 1 week before any procedures were carried out and were 9–11 weeks old at the start of the experiment. Normal human serum was obtained and pooled from five healthy adult volunteers according to an approval of the University of Graz Ethics Commission (GZ. 39/31/63 ex 2012/13). All volunteers provided written consent.

### Bacterial strains, plasmids and growth conditions

All bacterial strains and plasmids used in this study are listed in Supplementary Table 3. Unless stated otherwise, bacteria were grown at 37 °C with aeration in Luria–Bertani (LB) broth or on LB agar in the case of *E. coli* and *V. cholerae*, or in brain heart infusion (BHI) broth or on BHI agar supplemented with NAD and either protoporphyrin IX (PPIX) or hemin solution (stock-solution containing a mixture of hemin, L-histidine and triethanolamine) in the case of *H. influenzae*. BHI–NAD–PPIX served as standard growth medium for *H. influenzae*. BHI–NAD–hemin was used only for the following experiments: construction of transposon insertion mutants, deletion mutants and complementation strains; quantification of OMVs under conditions of iron excess or depletion; and quantitative real-time RT-PCR experiments. Supplements were used in the following final concentrations: NAD, 10 μg ml^−1^; hemin, 20 μg ml^−1^; L-histidine, 20 μg ml^−1^; triethanolamine, 0.08%; and PPIX, 20 μg ml^−1^. When appropriate, streptomycin (Sm, 100 μg ml^−1^), chloramphenicol (Cm, 2 μg ml^−1^), kanamycin (Km, 10 μg ml^−1^), or 2,2′-dipyridyl (100 μM) were added to *H. influenzae* growth media. *E. coli* and *V. cholerae* growth media were supplemented with streptomycin (Sm, 100 μg ml^−1^), kanamycin (Km, 50 μg ml^−1^), ampicillin (Ap, 100 or 50 μg ml^−1^ in combination with other antibiotics), sucrose (10%), arabinose (0.0002%), or isopropyl-β-D-thiogalactopyranoside (IPTG, 0.5 mM), if appropriate.

### Purification of OMVs and OM

For *H. influenzae*, OMVs as well as density gradient purified OMVs and OM were prepared as described previously^26^. *V. cholerae* and *E. coli* OMVs were isolated according to Schild *et al*.^10^ and Leitner *et al*.^11^, respectively. Briefly, OMVs were isolated from cultures grown to late exponential phase for 13 h (*H. influenzae*) or 8 h (*V. cholerae* and *E. coli*). Bacterial cells were removed from the supernatants containing the OMVs by centrifugation and subsequent filtration through 0.45 and 0.2 μm pore size filters. OMVs were pelleted from the filtrate by ultracentrifugation (144,000 × *g*, 4 h, 4 °C) using a Beckman Coulter Optima L-100 XP ultracentrifuge. In case of lipidome and proteome analyses as well as transmission electron microscopy, *H. influenzae* OMVs were further purified by density gradient ultracentrifugation using an isopycnic OptiPrep-iodixanol (Sigma-Aldrich) density gradient^26^. OM preparations were isolated from French press lysates obtained from *H. influenzae* cultures grown to late exponential phase for 13 h followed by purification steps via ultracentrifugation using a sucrose cushion and an isopycnic sucrose density gradient^26^. Protein concentrations of OMV and OM preparations were either determined by photometric TrayCell measurements of the absorbances at 260 and 280 nm using a Beckman Coulter DU730 spectrophotometer in combination with a TrayCell (Hellma) and the Warburg-Christian equation or by Bradford assays (Bio-Rad Laboratories, Protein Assay Dye Reagent) according to the manufacturer's manual. For all *H. influenzae*, *V. cholerae* or *E. coli* Bradford assays, an OMV preparation of Rd KW20, Vc AC53, or Ec BW whose protein concentration had been determined by a TrayCell measurement was used as a protein standard, respectively.

### SDS–PAGE and immunoblot analysis

The protein content of OMV and OM preparations was analysed by SDS–PAGE^42^ in combination with 12% polyacrylamide gels using the Prestained Protein Marker Broad Range (New England Biolabs) as a molecular mass standard. Protein bands were visualized by a colloidal Coomassie brilliant blue G250 staining according to Kang *et al*.^43^. Immunoblot analysis was performed as described previously^26^. The following primary antibodies and dilutions were used: anti-OMV mouse antiserum (see below), as well as anti-P1, -P2, -P4, -P5, or -P6 mouse antisera^26^ were diluted 1:500, the monoclonal antibody to the α-subunit of the *E. coli* RNA polymerase (NeoClone Biotechnology) was diluted 1:2,000. The horseradish peroxidase-conjugated goat anti-mouse IgG antibody (Dianova), diluted 1:7,500, served as secondary antibody. Chemiluminescence detection was performed by incubating each membrane in an ECL solution^26^ for 2 min with subsequent exposure in a ChemiDoc XRS system (Bio-Rad Laboratories) in combination with Quantity One software (Bio-Rad Laboratories).

### Generation of an antiserum against *H. influenzae* OMVs

To generate a specific polyclonal antiserum against *H. influenzae* OMVs, five female BALB/c mice were intraperitoneally immunized at days 0, 14 and 28 with approximately 2 μg of an OMV preparation derived from Rd KW20 dissolved in 0.1 ml PBS (pH 7.4). Sera were isolated from blood samples collected between day 38 and 46 as described previously^10^. Each serum was evaluated by immunoblot analysis against Rd KW20 OMVs before they were combined to generate the final polyclonal antiserum.

### Transposon mutagenesis and dot blot screen

Transposon mutagenesis in *H. influenzae* was accomplished according to a method described by Schlör *et al*.^44^. For this purpose, 480 independent transformations with pAKcat carrying the transposon Tn*10d-cat* were performed in Rd AK01. Cm-resistant colonies were pooled and extracted chromosomal DNA was cut with XmaI. The digested DNA was retransformed into Rd KW20 resulting in 2,820 chloramphenicol-resistant transposon insertion mutants, which were subsequently screened by dot blot for altered OMV production and amounts of the OMPs P1, P2, P4, P5 or P6 in OMVs. According to Akerley *et al*.^45^ the number of non-essential ORFs, which can be targeted by transposon mutagenesis is ∼1,300. Thus, the screen presented herein represents an at least twofold coverage, which may not achieve saturation. To this end, transposon insertion mutants were grown for 16 h in 200 μl of BHI–NAD–PPIX–chloramphenicol broth in quintuplicate in 96-well U-bottom plates (BD-falcon). The optical densities at 490 nm (OD_490_) were measured and cells were subsequently pelleted (2,000*g*, 10 min, room temperature). About 1 ml of pooled supernatant per transposon insertion mutant culture was filtered through a 0.2 μm pore size Supor (polyethersulfone) filter (Pall AcroPrep Advance 96-well filter plate) by centrifugation (2,000*g*, 2 min, room temperature) and each filtrate was stored at –20 °C until dot blots were performed. For dot blot analysis, filtrates were thawed and appropriate volumes were blotted onto six Amersham Hybond ECL nitrocellulose membranes (GE Healthcare) using a 96-well Whatman Minifold I Dot-Blot System (GE Healthcare). In addition, each membrane was spotted with the respective volume of a prior made and aliquoted wild-type (Rd KW20) filtrate (prepared in the same manner as the transposon insertion mutant filtrates) as a positive control and a sterile control filtrate as a negative control. Then, each membrane was dried, incubated in Tris-buffered saline (TBS) (20 mM Tris/HCl, pH 7.5, 150 mM NaCl) for 2 min, and blocked in 10% skim milk in TBS for 2 h. Afterwards, the six membranes were incubated with either anti-OMV, -P1, -P2, -P4, -P5, or -P6 mouse antiserum (primary antibodies, see above) diluted 1:500 in 10% skim milk in TBS overnight. The next day, membranes were washed three times in TBS for 10 min, incubated with the secondary antibody (horseradish peroxidase-conjugated goat anti-mouse IgG antibody from Dianova, diluted 1:10,000 in 10% skim milk in TBS) for 2 h, washed once in TBS-T (20 mM Tris/HCl, pH 7.5, 250 mM NaCl, 0,05% Tween-20), and twice in TBS for 10 min each. Finally, chemiluminescence detection was performed as described above. Relative intensities of dot blot signals derived from transposon insertion mutants were evaluated by eye through comparison with the respective wild-type signals in combination with the respective OD_490_ values. Representative examples for dot blots as well as the evaluation of relative signal intensities are provided in Supplementary Fig. 1. Interesting mutants with stronger or weaker OMV, OMP-P1, -P2, -P4, -P5 and/or -P6 signals compared with the wild-type were validated once. Then, chromosomal Tn*10d-cat* insertions were located by DNA sequencing of junction fragments generated by PCR using the transposon mutagenesis oligonucleotide primers, which are listed in Supplementary Table 4, and a previously described method^44^,^46^.

### Construction of deletion mutants and complementation strains

For construction of deletion mutants and complementation strains in *H. influenzae*, overlap extension PCRs in combination with a Rd KW20 transformation protocol were performed as described previously^26^. Corresponding oligonucleotide primers for amplification of up- and downstream fragments, the *cat* gene of pAKcat, and the *npt* gene of pACYC177, as well as primers for validation of correct construction are listed in Supplementary Table 4. *In cis* complementations were achieved by replacing the *cat* gene of a deletion mutant with the respective gene (upstream fragment containing upstream sequence and gene of interest) and the *npt* gene due to homologous recombination. For construction of deletion mutants in *V. cholerae*, isolation of chromosomal DNA, PCRs, purification of plasmids or PCR products, construction of suicide plasmids, as well as subsequent generation of deletion mutants were carried out as described previously^47^. For this purpose, the plasmid pCVD442, as well as the strains DH5αλpir, SM10λpir and Vc AC53 were used to construct the suicide plasmids pCVDΔ*vacJ*, pCVDΔ*yrbE*, pCVDΔ*fur*, as well as the respective deletion mutants. The expression plasmids pvacJ and pyrbE were constructed in a similar manner. PCR fragments of the respective genes containing their own ribosomal binding sites were generated using oligonucleotide primer pairs digested with the respective restriction enzymes indicated by the name of the oligonucleotide primer, and ligated into the similarly digested IPTG-inducible expression vector pMMB67EH. To construct expression plasmids for the *E. coli mlaF-B* genes, a PCR fragment containing the respective genes generated using oligonucleotide primer pairs digested with the respective restriction enzymes indicated by the name of the oligonucleotide primer, was ligated into the similarly digested IPTG-inducible expression vector pTRC99A. From there, a fragment containing the *mla* gene cluster was cut by NcoI and XbaI and ligated into a similarly digested pBAD24 to generate the arabinose-inducible expression plasmid pBADmlaF-B. Expression constructs were transformed into DH5αλpir, and Ap^r^ colonies were characterized by PCR. All mutants and plasmids described herein were confirmed by PCR (data not shown). Used oligonucleotide primers are listed in Supplementary Table 4.

### Quantification of OMVs

To compare the amount of OMVs produced by different *H. influenzae*, *V. cholerae* and *E.* coli strains, OMVs were purified essentially as described above with slight modifications. Cultures were grown to late exponential phase, OD were measured, and 70 ml (*H. influenzae* and *V. cholerae*) or 140 ml (*E. coli*) of the sterile-filtered supernatant was ultracentrifuged (150,000*g*, 4 h, 4 °C) using a Type 45 Ti rotor (Beckman Coulter). The pellet comprising the OMVs was immediately resuspended in 100–110 μl PBS (pH 7.4) and stored at –20 °C. To quantify the protein content of OMVs, Bradford assays as described above were used. To quantify the lipooligosaccharide or lipopolysaccharide content of OMVs, purpald assays were performed as described previously^48^ using 3-deoxy-D-*manno*-octulosonic acid (Kdo) (Sigma-Aldrich) as a standard. OMVs were quantified by back-calculating the respective protein and lipooligosaccharide/lipopolysaccharide content to 1 l original culture volume per OD unit (mg l^−1^ OD unit^−1^). In case of *H. influenzae* cultivated with BHI–NAD–PPIX an additional method for OMV quantification based on dot blot analysis as described above was used. To this end, the same volume of sterile-filtered supernatants derived from all strains to be tested were dot blotted on the same membrane and anti-OMV mouse antiserum was used as primary antibody. Dot blots were developed as described above and OMV signal intensities of dots were determined using the Quantity One software (Bio-Rad Laboratories) to calculate the respective artificial units (a.u.) per OD_490_ unit (a.u. OD_490_ unit^-1^).

### Size distribution and visualization of OMVs

Nanoparticle tracking analysis was used to measure the respective OMV size distribution of *H. influenzae*, *V. cholerae* or *E. coli* OMV preparations and transmission electron microscopy was applied for OMV visualization using *H. influenzae* density gradient purified OMV preparations as described previously^26^. Briefly, respective OMV preparations were examined via nanoparticle tracking analysis using a NanoSight LM10-HS instrument (Malvern Instruments) in combination with the nanoparticle tracking analysis software suite (version 2.3). For this analysis, a monochromatic laser beam at 405 nm was applied to the diluted suspension of OMVs and a video of 90 s duration was taken with a frame rate of 25 frames per s. Measurements were performed with ambient temperatures ranging from 22 to 24 °C. Particle movement was analysed by nanoparticle tracking analysis software with the minimal expected particle size, minimum track length and blur setting all set to automatic. Each video was then analysed to determine the respective mean and mode (particle size that appears most often within a given preparation) OMV size. To visualize *H. influenzae* OMVs by transmission electron microscopy, preparations were diluted to 0.1 mg ml^−1^ (protein equivalent) in PBS (pH 7.4) and 3 μl thereof was allowed to adsorb onto a carbon-coated copper grid for 15 s. After removal of excess liquid, samples were negatively stained with 2% uranyl acetate for 15 s followed by a further removal of excess liquid. Micrographs were recorded using a Morgagni 268 (FEI) transmission electron microscope.

### OM integrity assays

To compare the OM stability between different *H. influenzae* strains, the degree of cell lysis was assessed by dot blot analysis and minimal inhibitory concentrations of SDS and polymyxin B were determined. Dot blot analysis was performed as described above for the quantification of OMVs, except that a monoclonal antibody to the α-subunit of the *E. coli* RNA polymerase (RpoA, NeoClone Biotechnology, diluted 1:2,000) was used as primary antibody to detect RpoA in the sterile-filtered supernatants. RpoA detection has previously been used as a cytoplasmic contamination and lysis control in *H. influenzae*^26^. Minimal inhibitory concentrations were determined by using respective mid-log phase grown cultures to inoculate each well of a 96-well plate to a final OD_490_ of 0.1. Appropriate serial dilutions of SDS and polymyxin B were added and the plate was incubated at 37 °C for 16 h. Afterwards, the OD_490_ of each well was measured and compared with wells not containing bacteria (negative control). The minimal inhibitory concentration was defined as the lowest concentration of SDS or polymyxin B that inhibited bacterial growth (OD_490_<0.3).

### Proteome analysis

Mass spectrometry was used to identify proteins from density gradient purified OMV and OM preparations. Sample preparation, mass spectrometry, and protein identification by the Mascot search engine (version 2.3.02, Matrix Science) were performed as described previously^26^. Briefly, 0.7 mg (protein equivalent) of respective preparations were subjected to trypsin in-solution digestion. Therefore, samples were subjected to lipid extraction and resuspended in ammonium bicarbonate. Addition of DTT ensured reduction of disulphides in the samples, which were alkylated via iodoacetamide treatment. After trypsin digestion, samples were lyophilized, resuspended in 10 μl 0.1% trifluoroacetic acid, desalted using ZipTip C_18_ microcolumns (Millipore) according to the manufacturer's manual, and resuspended in 8 μl 0.1% formic acid. Finally, samples were analysed using a hybrid quadrupole orthogonal acceleration time-of-flight (Q-TOF) mass spectrometer (Waters) equipped with a nanoACQUITY ultra performance liquid chromatography system (Waters). The resulting MS/MS spectra were used for the identification of proteins by the Mascot search engine using the NCBInr database with the bacteria (eubacteria) taxonomy selected. Searches were carried out with the fixed modification carbamidomethyl (C), the enzyme selected as trypsin allowing up to 1 missed cleavages, a peptide tolerance of 1.2 Da, a MS/MS tolerance of 0.6 Da, a peptide charge of 1+, 2+ and 3+, monoisotopic mass values, and the instrument setting selected as ESI-QUAD-TOF. All data were searched with the significance threshold set to a *P* value of <0.05 resulting in an identity ion score threshold between 58 and 61. Only results with ion scores greater than or equal to the identity threshold were considered significant.

### Lipidome analyses

Lipids from density gradient purified OMV and OM preparations were extracted according to Folch *et al*.^49^ with minor modifications. For phosphatidylethanolamine composition analysis, lipids were extracted twice with a solvent containing 1% acetic acid, 500 nM butylated hydroxyl toluene, and 4 nM C17:0 phosphatidylcholine per sample as internal standard (ISTD). For determination of FAs, PLs, and phosphatidylethanolamine composition, lipids were extracted from samples containing 300 μg, 1,000 μg, and 50 μg protein, respectively. All lipid extracts were dried under a stream of nitrogen and stored at –20 °C until analysis. For FA analysis, dried lipid extracts were transesterified to fatty acid methyl esters (FAMEs) by methanolysis containing 2.5% sulphuric acid at 85 °C for 90 min. FAMEs were extracted into light petroleum ether/water (3/1, vol/vol) and analysed by gas liquid chromatography and flame-ionization detection (Hewlett-Packard 6890) using a HP-INNOWax capillary column (15 m × 0.25 mm i.d. × 0.50 μm film thickness) with helium as carrier. FAME species were identified by comparison to the FAME standard mix GLC-68B (Nu-Chek, Inc) and C26:0 FAME (Sigma-Aldrich). For PL analysis, lipid extracts and, for comparison, a PL mix were separated by one-dimensional thin-layer chromatography on Silica gel 60 plates (Sigma-Aldrich) using chloroform/acetone/methanol/acetic acid/water (50/20/10/10/5, per vol.). Lipids were stained with iodine vapour, stained spots were scraped off and phosphate residues of PLs were liberated by treatment with H_2_SO_4_ (72%)/HClO_4_ (9/1, vol/vol), which were subsequently quantified colourimetrically as phosphomolybdate measured at 820 nm according to the standard procedure described by Broekhuyse^50^. For phosphatidylethanolamine species analysis, dried lipids were dissolved in 2-propanol/chloroform/methanol (7/2/1, per vol.) and analysed by LC/ESI-MS using an Acquity UPLC HSS T3 column (100 Å, 1.8 μm, 2.1 × 100 mm, Waters)^51^. Extraction efficacy and lipid recovery were normalized using ISTD.

### Quantitative real-time RT-PCR

Expression of *vacJ, yrbE*, *fabB*, *fabD* or *fabH* was determined by quantitative real-time RT-PCR (qRT-PCR). For this purpose, respective strains were grown to an OD_490_ of 0.8-1 in BHI–NAD–hemin medium supplemented with or without 100 μM 2,2′-dipyridyl in case of *H. influenzae* or to an OD_600_ of 0.7–0.9 in LB medium in case of *V. cholerae* and *E. coli*. Bacterial RNA extraction, DNase digestion, cDNA synthesis and qRT-PCR were performed as described previously^52^. To determine the gene expression of *vacJ* and *yrbE* in *H. influenzae* under *in vivo* conditions, CD-1 mice were intranasally inoculated with Rd KW20 essentially as described previously^8^ with an inoculum of approximately 5 × 10^8^ c.f.u. per mouse. After 4 h, the mice were sacrificed, the nasopharynx of each mouse was removed by dissection and mechanically homogenized in 2 ml Trizol. RNA was subsequently extracted using chloroform extraction and precipitated with isopropanol. DNase digestion, cDNA synthesis and qRT-PCR were performed as described previously^53^. Corresponding oligonucleotide primers are listed in Supplementary Table 4. Relative gene expression comparisons were obtained through the ΔΔC_T_ method by normalizing the mean cycle threshold of each investigated transcript to the housekeeping gene *rpoB* and to one randomly selected reference sample.

### Serum bactericidal assay

To test *H. influenzae* strains in serum bactericidal assays, cells were grown to an OD_490_ of 0.8–1 and diluted in Hank's buffer (HBSS from Gibco, with Ca^2+^ and Mg^2+^, no phenol red) to an OD_490_ of 0.1. About 100 μl of this suspension were mixed with 5 μl normal human serum (2% final concentration) and optionally with 5 μl of appropriate dilutions of respective OMV preparations. This resulted in the following physiological final *in vitro* concentrations of OMVs based on the OMV quantification via purpald assays (lipooligosaccharide equivalent) of ∼0.6 mg l^−1^ OD_490_ unit^−1^ for Rd KW20-derived OMVs and 1.2 mg l^−1^ OD_490_ unit^-1^ for Rd Δ*vacJ* derived OMVs. In case of two-fold amounts of Rd KW20-derived OMVs, 10 μl were added, which result in a final concentration of approximately 1.2 mg l^−1^ OD_490_ unit^−1^. Hank's buffer was added beforehand to obtain a final reaction volume of 250 μl. The mixture was incubated at 37 °C for 45 min with rotation, the assay was stopped by cooling to 4 °C, and appropriate dilutions were plated on agar plates to determine viable counts. To calculate the percentage of survival, viable counts were compared with control samples in absence of normal human serum.

### Statistical analysis

Data were analysed using GraphPad Prism version 6.0f for Mac OS X (GraphPad Software). The statistical significance of differences between groups was examined using the unpaired t test or an ordinary one-way ANOVA followed by Sidak's multiple comparison post test. Differences were considered significant at *P* values of <0.05.

## Additional information

**Accession codes:** The mass spectrometry proteomics data have been deposited to the ProteomeXchange Consortium via the PRIDE partner repository with the data set identifier PXD003249.

**How to cite this article:** Roier, S. *et al*. A novel mechanism for the biogenesis of outer membrane vesicles in Gram-negative bacteria. *Nat. Commun.* 7:10515 doi: 10.1038/ncomms10515 (2016).

## Supplementary Material

Supplementary Information Supplementary Figures 1-6, Supplementary Tables 1-4 and Supplementary References
