# Supplementary material for: A novel mechanism for the biogenesis of outer membrane vesicles in Gram-negative bacteria
Source: Nat Commun. 2016 Jan 25;7:10515. doi: 10.1038/ncomms10515 (PMC4737802; doi:10.1038/ncomms10515)
Supplement: Supplementary Information — Supplementary Figures 1-6, Supplementary Tables 1-4 and Supplementary References [file ncomms10515-s1.pdf]

SUPPLEMENTARY FIGURES

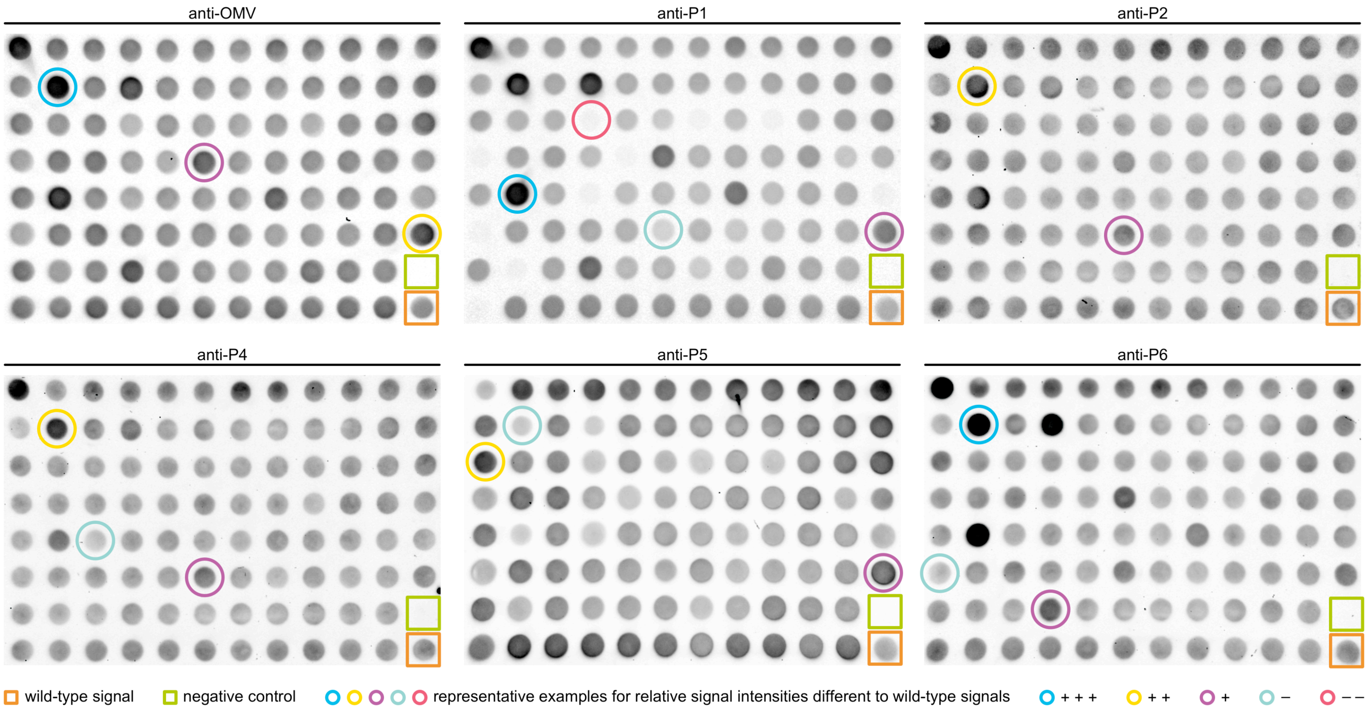

Supplementary Figure 1 | Representative examples of the dot blot screening. Shown are representative examples of dot blots incubated with anti-OMV, -P1, -P2, -P4, -P5, or -P6 mouse antiserum. Each dot blot was spotted with transposon insertion mutant filtrates, a wild-type filtrate as a positive control, and a sterile control filtrate (BHI-NAD-PPIX broth) as a negative control. For details please see the respective method section (transposon mutagenesis and dot blot screen). Relative intensities of dot blot signals derived from transposon insertion mutants were evaluated by eye through comparison with the respective wild-type signals in combination with the respective OD_{490} values. Colored circles highlight representative examples of relative signal intensities different to wild-type signals. Increased relative signal intensities are marked by +, + +, or + + + (in ascending order), whereas decreased relative signal intensities are given by – or – – (in descending order).

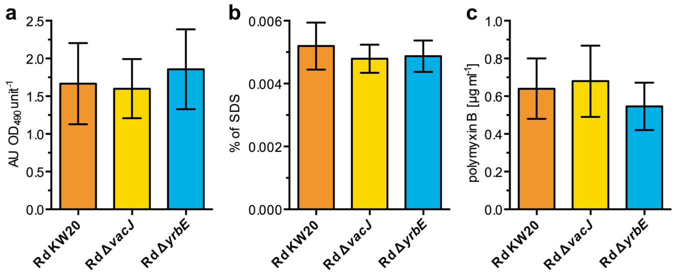

Supplementary Figure 2 | PL transporter mutants reveal intact OM integrity in *H. influenzae*. (**a**) Cell lysis of Rd KW20, Rd Δ*vacJ*, and Rd Δ*yrbE* was investigated by dot blot analysis. Mean values with s.e.m. are shown (n=6 biological replicates). (**b**, **c**) Mean minimal inhibitory concentrations with s.e.m. of SDS (**b**) and polymyxin B (**c**) are shown (n=5 biological replicates).

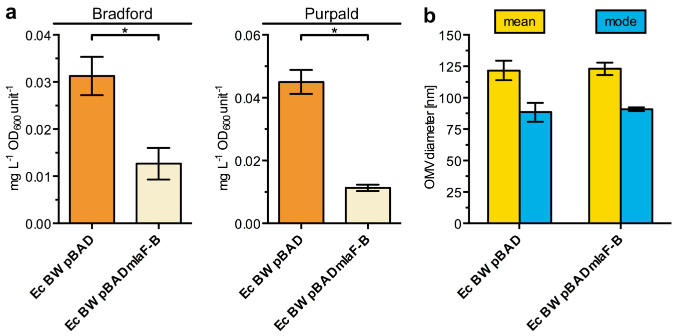

Supplementary Figure 3 | Overexpression of the *mla* gene cluster results in decreased OMV production in *E. coli*. (**a**) OMV preparations derived from equivalent OD_{600} units of cultured wild-type containing vector control (Ec BW pBAD) or expression plasmid (Ec BW pBADmlaF-B) were analyzed for total protein (Bradford) and lipopolysaccharide (Purpald). Mean values with s.e.m. are shown (n=6 biological replicates). Significant differences between the data sets are marked by asterisks (P<0.05; unpaired t test). (**b**) Distributions of OMV sizes produced by the strains were determined by nanoparticle tracking analysis. Mean values with s.d. of mean and mode OMV diameter sizes within respective OMV preparations are shown (n=6 biological replicates).

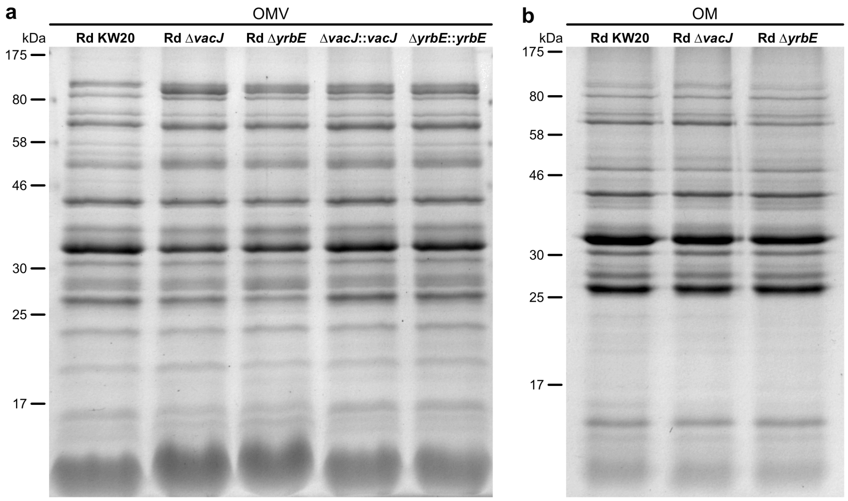

Supplementary Figure 4 | OMV and OM protein profile comparisons between *H. influenzae* strains. (**a**, **b**) The protein profiles of OMV (**a**) or OM (**b**) preparations derived from Rd KW20, Rd Δ*vacJ*, Rd Δ*yrbE*, Rd Δ*vacJ*::*vacJ*, and Rd Δ*yrbE*::*yrbE* were compared by SDS-PAGE. Approximately 8 μg protein equivalent of each sample was used for the analysis. Lines to the left indicate the molecular masses of the protein standards in kDa.

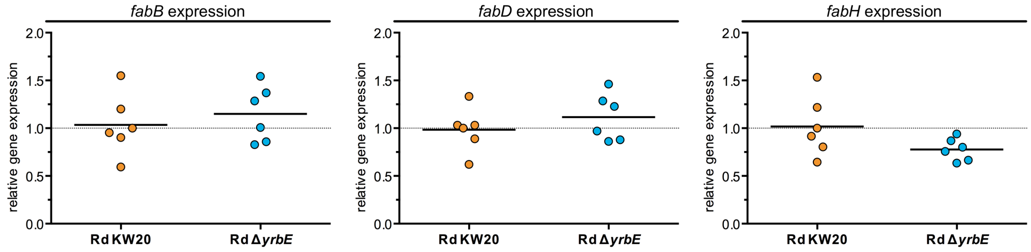

Supplementary Figure 5 | PL accumulation in OMVs derived from *H. influenzae* PL transporter mutant Rd Δ*yrbE* is not a result of increased expression of fatty acid biosynthesis genes. Relative expressions of *fabB*, *fabD*, and *fabH* in Rd KW20 and Rd Δ*yrbE* grown in BHI-NAD-hemin were determined by qRT-PCR. Horizontal bars highlight the mean of each data set (n=6 biological replicates). Dotted lines indicate a relative gene expression of 1.

Haemophilus influenzae

H. influenzae HI_0718 (*vacJ*)
TGCACCTATTTCCAAACGTGAAGCGGATACTATTTTAAATCGTCTTGAACAAAATACTGATAAACCTCGTCATCGTAATGAATATCATCCGGGAGAGGAAGTTCGT
GTGACTGAAGGGCCATTTGCTGATTTTAATGGTACAGTAGAAGAAGTGGATTATGAAAAAGGCCGCTTAAAAGTGTCTGTATCAATGTTTGGCCGTGCTACACCAG
TTGAGCTTGAATTTGGTCAAGTTGAAAAAATACACTAAGAAATGATTAAAATATAACCGCACTTTTGTAGTGCGGTTTTTTGTTTTTAATGTTTGTTGAAACAGCA
TAAAAGAGTGT**T**TTTATACACAAAATTTGTTGTTATTTCACAAATTAGGTAGAATAATCCCATATTTATGTAAGGATTAAAAATG

H. influenzae HI_1087 (*yrbF*)
GTTTTTCTACTTTCCTCTCTATAACTTTCCTCTCTATAACTTTCCTCTCTATAACTTTTCTCTTGCAATCTTTTTTATCAAATCTGTATCTTTTACAGGCTTATTA
TTTATAAGCATTTATCAATGTTTATTTTCCATAAACAAAGTATTATTAAAATTCAATAAATAATGAAGAAATGTAAAGCTTCTTTAGTGAGGTCAAAAAGAATGTT
ACTATTACAAAAA**T**TTTAATCTATAAAAAATAGAAATAAAATTTATC**A**CTATTTTTATGACCACATCAAAGTTAAACCAAAGAGATGTGTTGCGATAAAAGAAAAA
ACTTCATCTTTAAATTAAACAAATCTAACTTTTTCAGGTCATATAAAACTGATTGAACTTTTTATTTATAAACGAGATATTAATG

Vibrio cholerae

V. cholerae VC_2048 (*vacJ*)
GAGGGCATGGCAGAGGGTCAATCGGTGAAAGTCACCATTAATCTAGGCGAGCAGGTTAAGGTTGATCCTAACGCAGTGTTGATCGTATCTATCCATGCTCCCGGTT
CGCCAATGCCTGTTGCTGCAGCCCGTTATCCGCTAGGTGAGTTTCCAAGAACCGTGGTGTTGGATGACAATAACAGCATGATGGAAGGATCAAAATTGTCCAGTCT
TGAGTCGTATGTGGTACGTGTGCGTATCGACAGCGATGGTAATGTGGCGACGAAAAGCGGTGACTGGTATGCCGAAAGTCCCACAATGAAAACAGGTGAGCCTGTT
GACGTTGTGCTTGATAAGCAGTATGAATAAAATCACAAGCTGAAAAATAATATAACAA**T**AAGGCCGATGGGATCATCGGCCTTTG

V. cholerae VC_2520 (*yrbF*)
GACTCTTAGAGCAAGGCAAACACCTCCATCAATGGACGAATTCACGCAAGGTCAGGCATCTTCAGCCATTTAGGCAGAGAATTGGCCGCCAATTTTGACGTGTTGG
GTATTAAAAGGGAAGTCGAAGTTCCAATTAATTATCCTTATCCCCTTGCGATTTTGCCTGTTTGTCACAGTAAACGTCAGCCTTTAAAGCTTGTTCCTAGGTTGTC
GTCGGCATGCTATCGACTATGCTGGATGAAGACTTTTATCGGGTTTTTAGACGCAAAATGAGTTGAACGACTTTGACAATTCTTGGTCAAGAACCCAAGCATGGAT
TGATGCGTGCTTTTAATTGCACCATATTGTGCA**T**TATCATGCGCATTCTTTCAGGATGTCGACCTAACAGTGAAGTGCCCATATG

Escherichia coli

E. coli b_2346 (*vacJ*)
CGGGAGGGGAGTCGGTCTTCATCTATTTCTATTTTTTTGCCGCGCTCTTTTTCTTCGCTCTCAACTTCAATTTCGTCGCTGTGTTGATCAATTTTGTCGTTGTCCA
TGGTCTCTTCTCTTGAATTAGCACGTATAGCTAAAGCGTAGCGGCTTTTTTGCTCGCAACTGACGGGAGTTACTCTGAAAATGTAGAAAAGGCTGCGTTTGCCTTT
TTCTGTTTCTATAGAATCAAGTAGCCTACAGGGCGGCGATTACCAGGCTATGATCAAATCAGCAAATCAGGGCGTCTGGACATCAGTTGACGTGCTGTTACAATCG
CCC**A**CACCTAAACAGGCGGATACGGTATCGTTCCGTCATGGATGGCAAACTGCATAAGCCATAAAAAAACAGGGAGACATTTATG

E. coli b_3195 (*yrbF*)
TCGTGCAGAGACGCGGCAAGCGAGACGATGACTTCTGGTAACGATGTACCAATACTGACCACCGTCATGCCGATGATCAGCGGCGGGATGCCAAAGGTTCGGCAAA
GAATAGACGCGGCAAAAACCAGGCGGTCGGCACTGTAAACGACCAAAAGTAAACCAACAATTAACAGTGCCGTAGCTAAAAGCATCTAACGTCCTTTCTTCAGGTA
TACTCGCCGGTCCGCTGAAGATTTTCAGAAAGCCGTAACGGATGCTTAATTTTGACT**T**TATGCGGCTAAAAAGTAAAACAAATGCCAGCTTTCGCTAACCACGGCG
GGTAATATTCTGTAAATATGTTGGGTTCAAGGTTAAATTGAGCGCCATGCTTAGAAAATCAACGCAAGACGAAGGGTGAATTATG

Supplementary Figure 6 | *In silico* analyses of potential Fur binding sites in upstream regions of *vacJ* and the *yrb* gene cluster. The 400 nucleotide regions upstream of the translational start codons (highlighted in green) of *vacJ* and *yrbF* are shown for *H. influenzae*, *V. cholerae*, and *E. coli*, respectively. The promoter regions according to Neural Network Promoter Prediction[1] are indicated in yellow with the +1 transcriptional start site in bold. Please note that in case of the *yrb* gene cluster of *H. influenzae* two promoters with equal scores were identified. Consequently, both are highlighted in yellow and two +1 transcriptional start sites are shown in bold. The putative areas containing Fur binding sites according to EMBOSS Needle[2-5] are underlined and were obtained from Harrison *et al.*[6] for *H. influenzae,* Escolar *et al.*[7] for *E. coli*, and Mey *et al.*[8] for *V. cholerae* using the best or second best conserved nucleotide, if applicable.

SUPPLEMENTARY TABLES

Supplementary Table 1: Transposon insertion mutant phenotypes detected by dot blot screening.

Gene no.[*]	Description[*]	Insertion[†]	Relative signal intensity[‡]					
			OMV	P1	P2	P4	P5	P6
HI_0037	Rod shape-determining protein MreB	dn 993-1019	+ +	+ +	+	+	+ +	+
HI_0401	Outer membrane protein P1	dn 315	–	– –				
HI_0457	Hypothetical protein HI0457	up 40	+	+	+		+	
HI_0528	Tyrosine-specific transport protein TyrP	dn 953	+ +	+ +	+	+	+ +	+
HI_0572	Peroxiredoxin hybrid Prx5	dn 10-38	– –	–	–	–	–	–
HI_0854	Heme iron utilization protein	up 65	–	–	–	–	–	–
HI_0980	DNA-binding protein Fis	dn 250	+				+	
HI_1083	NTP binding protein YrbB	dn 92-115	+ +	+	+		+	+
HI_1086	ABC transporter permease YrbE	dn 345	+ +	+	+	+	+ +	+
HI_1099	Hypothetical protein HI1099	dn 274	+		+		+	+
HI_1164	Outer membrane protein P5	dn 657	+ + +	+ + +	+	+	–	+ + +
HI_1181	Phosphoheptose isomerase GmhA	dn 140	+ +	+ +	+	+		+
HI_1213	Thiol-disulfide interchange protein DsbC	dn 607	+ + +	+ +	+	+	+ + +	+
HI_1251	Virulence-associated protein A	dn 194-208	+				+	
HI_1441	Stringent starvation protein A	dn 299-316	–					
HI_1587	DNA-binding protein H-NS	up 9	+				+	
HI_1676	Molybdenum cofactor biosynthesis protein A	dn 458-470	+			–	+	–
HI_1683	Na(+)-translocating NADH-quinone reductase subunit E	up 162	+				+	
HI_1702	Methyltransferase MetE	dn 47-1537	+	+	+		+	
HI_1727	Argininosuccinate synthase ArgG	dn 488-1259	+				+	+

[*]Identified gene disruptions that lead to altered OMV production and amounts of the OMPs P1, P2, P4, P5, or P6 in OMVs are ordered by their corresponding gene numbers, which are listed along with a description of their normal gene products.

[†]Transposon insertion sites of disrupted genes are given by the number of base pairs upstream (up) or downstream (dn) of the respective translational start site. In the event that several transposon insertions within a gene are leading to the same or a similar phenotype, the first and the last insertion site are given separated by a hyphen.

[‡]Relative intensities of dot blot signals derived from transposon insertion mutants were evaluated by eye through comparison with the respective wild-type signals in combination with the respective OD_{490} values. Relative signal intensities represent mean phenotypes of two independent experiments per transposon insertion mutant. If applicable, the mean phenotypes of mutants with identical or different transposon insertions within the same gene were averaged. Increased relative signal intensities are marked by one, two, or three plus signs (in ascending order), whereas decreased relative signal intensities are highlighted by one or two minus signs (in descending order). A blank field indicates no visible differences compared to wild-type signals. Representative examples for dot blots as well as examples for the evaluation of relative signal intensities are provided in Supplementary Fig. 1.

Supplementary Table 2: OMV and OM proteins derived from Rd KW20, Rd Δ*vacJ*, and Rd Δ*yrbE* identified by mass spectrometry.

Identified protein[*]	Accession no.	Gene no.	kDa[†]	Loc.[‡]	OMV[§]			OM[§]		
					Rd KW20	Rd Δ*vacJ*	Rd Δ*yrbE*	Rd KW20	Rd Δ*vacJ*	Rd Δ*yrbE*
Outer membrane protein 26	gi|16272853	HI_0916	22	OM	727-1	436-8	399-8	120-15	175-9	234-11
Outer membrane protein P6	gi|16272329	HI_0381	16	OM	716-2	501-6	374-9	325-3	217-5	334-5
Outer membrane protein P2	gi|16272106	HI_0139	39	OM	706-3	976-1	1008-1	771-1	523-1	1110-1
Galactose ABC transporter, periplasmic-binding protein MglB	gi|1573835	HI_0822	38	PP	700-4	892-2	900-2	133-11	227-4	240-10
Hypothetical protein HI1492	gi|16273393	HI_1492	19	U	643-5	567-4	455-7	–	–	–
Oxidoreductase, thiol:disulfide interchange protein DsbA	gi|16272787	HI_0846	23	PP	625-6	639-3	548-5	–	–	–
High-affinity zinc transporter substrate-binding protein ZnuA	gi|16272089	HI_0119	38	PP	450-7	457-7	502-6	96-21	164-11	277-8
D-methionine-binding lipoprotein MetQ	gi|16272562	HI_0620	30	IM	420-8	261-15	178-23	88-25	139-13	146-31
Serine protease HtrA	gi|16273177	HI_1259	49	PP	337-9	336-9	326-10	–	87-28	157-27
Sialic acid-binding periplasmic protein SiaP	gi|16272113	HI_0146	37	PP	317-10	292-12	231-15	–	–	–
D-ribose transporter subunit RbsB	gi|16272448	HI_0504	30	PP	286-11	560-5	567-4	–	–	–
Translocation protein TolB	gi|16272330	HI_0382	45	U	282-12	305-10	195-20	116-17	105-19	171-24
Thiol-disulfide interchange protein DsbC	gi|16273132	HI_1213	25	PP	281-13	165-25	155-26	–	–	–
Arginine ABC transporter substrate-binding protein	gi|16273101	HI_1179	26	PP	246-14	296-11	320-11	–	83-31	88-76
Hypothetical protein HI1505	gi|16273407	HI_1505	34	CP	245-15	–	116-38	–	–	–
FKBP-type peptidyl-prolyl cis-trans isomerase FkpA	gi|16272517	HI_0574	26	PP	214-16	237-17	151-27	–	–	–
Protein D, glycerophosphodiester phosphodiesterase	gi|16272631	HI_0689	42	OM	198-17	274-14	181-21	98-20	79-33	161-26
Antigen, penicillin-binding protein activator LpoA	gi|16273542	HI_1655	64	OM	198-18	291-13	610-3	–	–	–
Thiamin ABC transporter substrate-binding protein TbpA	gi|16272953	HI_1019	37	PP	195-19	88-37	112-41	–	–	–
Hypothetical protein HI1681	gi|16273568	HI_1681	23	U	184-20	228-18	108-44	92-22	111-17	186-21
Iron-utilization periplasmic protein hFbpA	gi|30995354	HI_0097	36	PP	179-21	213-21	242-13	–	–	–
Peptidyl-prolyl cis-trans isomerase	gi|30995417	HI_1004	70	IM	176-22	96-35	101-47	105-19	114-16	204-17
Outer membrane protein P1	gi|16272350	HI_0401	49	OM	163-23	215-20	212-17	162-8	301-2	373-4
Hypothetical protein HI1427	gi|16273332	HI_1427	31	U	159-24	104-33	206-18	–	61-59	112-47
Opacity protein (HI1174)	gi|30995429	HI_1174	17	OM	156-25	89-36	–	66-40	–	–
Outer membrane protein P4	gi|16272635	HI_0693	30	OM	140-26	167-23	196-19	171-7	171-10	259-9
L-asparaginase II	gi|16272686	HI_0745	37	PP	138-27	146-27	123-36	–	–	–
Lipoprotein, outer membrane protein assembly factor BamC	gi|16272214	HI_0256	24	OM	112-28	167-24	174-24	–	–	96-62
Hypothetical protein HI0449	gi|16272397	HI_0449	20	U	111-29	187-22	69-57	–	61-58	65-109
Hypothetical protein HI1709	gi|16273596	HI_1709	13	U	108-30	158-26	106-45	–	–	–
Spermidine/putrescine ABC transporter substrate-binding protein (HI1344)	gi|16273254	HI_1344	42	PP	107-31	243-16	240-14	126-12	–	115-43
Bifunctional 2',3'-cyclic nucleotide 2'-phosphodiesterase/3'-nucleotidase	gi|16272527	HI_0583	73	PP	102-32	85-38	137-31	–	–	–
Elongation factor Tu	gi|16272522	HI_0578	43	CP	101-33	67-48	76-55	122-13	77-37	113-45
Outer membrane protein P5	gi|16273088	HI_1164	38	OM	93-34	80-43	214-16	358-2	258-3	549-3
Small protein A	gi|542123256	HI_0838	16	OM	90-35	77-44	132-33	–	–	105-54
Hypothetical protein HI0973	gi|16272910	HI_0973	31	OM	89-36	124-30	149-28	–	–	–
Branched chain amino acid ABC transporter substrate-binding protein	gi|16273512	HI_1624	26	U	85-37	64-51	133-32	–	–	–
Trigger factor	gi|16272653	HI_0713	48	CP	84-38	–	60-60	–	–	85-78
Amino acid ABC transporter substrate-binding protein	gi|16273008	HI_1080	28	PP	79-39	226-19	282-12	–	–	–
Hypothetical protein HI0177, outer membrane protein assembly factor BamD	gi|16272142	HI_0177	29	OM	76-40	143-28	168-25	221-5	185-8	154-28
Oligopeptide ABC transporter substrate-binding protein	gi|16273049	HI_1124	61	PP	75-41	–	–	–	–	–
Opacity protein (HI1457)	gi|16273363	HI_1457	19	OM	69-42	74-45	112-39	–	–	–
50S ribosomal protein L7/L12	gi|16272584	HI_0641	12	U	68-43	–	–	73-34	60-60	104-56
Bacteriophage Mu I protein	gi|16273406	HI_1504	39	CP	64-44	–	–	–	–	–
Protective surface antigen D15	gi|16272854	HI_0917	87	OM	62-45	126-29	84-52	257-4	206-6	315-6

Molybdenum ABC transporter substrate-binding protein	gi|16273580	HI_1693	27	PP	61-46	85-39	–	–	–	–
Ferric ABC transporter protein	gi|16272098	HI_0131	38	U	58-47	–	179-22	–	–	–
NAD nucleotidase	gi|16272169	HI_0206	66	U	–	119-31	94-49	–	–	128-33
ABC transporter, periplasmic binding protein YrbC	gi|16273012	HI_1084	25	U	–	114-32	92-50	–	–	74-95
Heme-hemopexin utilization protein A	gi|16272222	HI_0264	99	EC	–	98-34	–	–	–	–
Iron-chelated ABC transporter substrate-binding protein	gi|16272311	HI_0362	32	PP	–	83-40	77-54	–	–	94-66
Stationary-phase survival protein SurA	gi|16272406	HI_0458	35	PP	–	82-41	111-42	60-48	–	–
Hypothetical protein HI1658	gi|16273545	HI_1658	20	PP	–	80-42	124-35	120-14	93-26	213-15
Molecular chaperone DnaK	gi|16273156	HI_1237	68	CP	–	68-46	99-48	61-46	78-35	153-29
D-xylose transporter subunit XylF	gi|16273036	HI_1111	36	PP	–	68-47	117-37	–	–	–
Hypothetical protein HI0930	gi|16272867	HI_0930	22	EC	–	67-49	88-51	–	–	–
Heme/hemopexin-binding protein B	gi|16272221	HI_0263	63	OM	–	65-50	–	–	61-57	–
DNA-binding protein HU-alpha	gi|30995375	HI_0430	9	CP	–	64-52	109-43	–	122-15	229-13
Immunoglobin A1 protease	gi|16272928	HI_0990	186	OM	–	–	145-29	72-35	–	84-79
Transferrin-binding protein	gi|16273136	HI_1217	103	OM	–	–	139-30	136-10	144-12	294-7
15 kDa peptidoglycan-associated lipoprotein	gi|16273474	HI_1579	15	OM	–	–	132-34	79-30	81-32	116-42
Phosphopyruvate hydratase	gi|16272869	HI_0932	46	CP	–	–	112-40	–	–	209-16
Lipoprotein NlpI	gi|16272193	HI_0230	36	IM	–	–	104-46	–	–	–
Spermidine/putrescine ABC transporter substrate-binding protein (HI0498)	gi|30995379	HI_0498	40	PP	–	–	79-53	–	–	–
Hypothetical protein HI1008, competence protein ComEA	gi|16272943	HI_1008	12	IM	–	–	74-56	–	–	–
Hypothetical protein HI0205	gi|16272168	HI_0205	29	U	–	–	62-58	–	–	–
Hypothetical protein HI1512	gi|16273414	HI_1512	13	U	–	–	61-59	–	–	–
Probable hemoglobin and hemoglobin-haptoglobin-binding protein 4	gi|19864326	HI_1565/1567	114	OM	–	–	–	178-6	73-43	147-30
Heme-binding lipoprotein	gi|16272793	HI_0853	61	PP	–	–	–	160-9	188-7	618-2
Hemin receptor	gi|16272087	HI_0113	85	OM	–	–	–	118-16	67-48	132-32
L-lactate dehydrogenase	gi|16273624	HI_1739.1	42	CP	–	–	–	106-18	100-22	190-19
HflK protein	gi|16272119	HI_0151	46	CP	–	–	–	89-23	65-51	109-51
30S ribosomal protein S1	gi|16273139	HI_1220	60	CP	–	–	–	88-24	96-24	118-39
30S ribosomal protein S10	gi|161350025	HI_0776	12	CP	–	–	–	84-26	65-50	92-69
Na(+)-translocating NADH-quinone reductase subunit A	gi|16272131	HI_0164	49	CP	–	–	–	84-27	97-23	170-25
Dihydrolipoamide acetyltransferase	gi|16273151	HI_1232	59	CP	–	–	–	84-28	–	–
F0F1 ATP synthase subunit B	gi|16272430	HI_0483	17	IM	–	–	–	81-29	107-18	123-36
Outer membrane protein	gi|16272337	HI_0389	21	OM	–	–	–	78-31	–	–
50S ribosomal protein L6	gi|16272734	HI_0793	19	CP	–	–	–	78-32	–	–
F0F1 ATP synthase subunit alpha	gi|16272428	HI_0481	56	CP	–	–	–	77-33	–	–
Lipoprotein VacJ	gi|16272658	HI_0718	28	OM	–	–	–	72-36	–	76-94
Preprotein translocase subunit SecD	gi|16272200	HI_0240	67	IM	–	–	–	70-37	–	86-77
Galactose/methyl galactoside transporter ATP-binding protein MglA	gi|16272764	HI_0823	57	IM	–	–	–	70-38	94-25	82-82
50S ribosomal protein L28	gi|16272889	HI_0951	9	CP	–	–	–	66-39	63-54	67-106
Lipoprotein (HI0162)	gi|16272129	HI_0162	22	U	–	–	–	65-41	–	–
Preprotein translocase subunit YajC	gi|16272201	HI_0241	11	IM	–	–	–	64-42	–	–
30S ribosomal protein S3	gi|16272724	HI_0783	26	CP	–	–	–	63-43	–	74-96
Thiamine biosynthesis lipoprotein	gi|16272137	HI_0172	38	U	–	–	–	62-44	–	–
Chaperonin GroEL	gi|16272487	HI_0543	58	CP	–	–	–	62-45	75-39	–
Extragenic suppressor, Inositol monophosphatase SuhB	gi|16272874	HI_0937	29	CP	–	–	–	61-47	–	89-74
Heme biosynthesis-like protein	gi|30995388	HI_0603m	45	U	–	–	–	–	125-14	186-20
Na(+)-translocating NADH-quinone reductase subunit C	gi|16272133	HI_0167	27	U	–	–	–	–	104-20	121-37
Hypothetical protein HI0370	gi|16272318	HI_0370	23	U	–	–	–	–	100-21	–
50S ribosomal protein L5	gi|542123255	HI_0790	20	CP	–	–	–	–	89-27	200-18
Hypothetical protein HI0442	gi|30995376	HI_0442	12	U	–	–	–	–	85-29	117-41
Pyruvate dehydrogenase subunit E1	gi|16273152	HI_1233	99	CP	–	–	–	–	85-30	–

Hypothetical protein HI1462, outer membrane efflux protein	gi|16273365	HI_1462	50	OM	–	–	–	–	78-34	215-14
NAD(P) transhydrogenase subunit alpha	gi|16273272	HI_1362	55	IM	–	–	–	–	77-36	233-12
Phosphomannomutase	gi|30995397	HI_0740	60	CP	–	–	–	–	76-38	108-52
Anaerobic dimethyl sulfoxide reductase subunit B	gi|16272978	HI_1046	23	IM	–	–	–	–	75-40	–
Phosphate acetyltransferase	gi|16273123	HI_1203	77	CP	–	–	–	–	75-41	124-35
Organic solvent tolerance protein, LPS-assembly protein	gi|16272671	HI_0730	90	OM	–	–	–	–	73-42	103-57
Single-stranded DNA-binding protein	gi|16272208	HI_0250	19	CP	–	–	–	–	70-44	–
Polynucleotide phosphorylase/polyadenylase	gi|16272192	HI_0229	77	CP	–	–	–	–	70-45	98-60
30S ribosomal protein S6	gi|16272491	HI_0547	14	CP	–	–	–	–	69-46	94-68
30S ribosomal protein S7	gi|16272524	HI_0580	18	CP	–	–	–	–	68-47	98-61
LexA repressor	gi|30995398	HI_0749	23	CP	–	–	–	–	66-49	–
Hypothetical protein HI1628	gi|16273517	HI_1628	15	IM	–	–	–	–	65-52	66-108
50S ribosomal protein L24	gi|16272730	HI_0789	11	CP	–	–	–	–	63-53	184-23
Acetolactate synthase 3 regulatory subunit	gi|16273478	HI_1584	18	CP	–	–	–	–	63-55	63-112
Nitrate/nitrite response regulator protein NarP	gi|16272666	HI_0726	23	CP	–	–	–	–	62-56	–
Sec-independent translocase TatB	gi|16273632	HI_0187b	20	IM	–	–	–	–	60-61	80-87
Universal stress protein A	gi|16272756	HI_0815	16	CP	–	–	–	–	60-62	95-64
Hypothetical protein HI0150, membrane protease subunit HflC	gi|16272118	HI_0150	33	CP	–	–	–	–	–	185-22
Ferric transporter ATP-binding protein	gi|16272096	HI_0126	36	IM	–	–	–	–	–	125-34
30S ribosomal protein S4	gi|16272743	HI_0801	24	CP	–	–	–	–	–	120-38
Protoheme synthesis protein HemY	gi|16272545	HI_0602	49	IM	–	–	–	–	–	118-40
50S ribosomal protein L2	gi|16272721	HI_0780	30	CP	–	–	–	–	–	114-44
Inner membrane protein translocase component YidC	gi|16272937	HI_1001	61	IM	–	–	–	–	–	113-46
DNA-directed RNA polymerase subunit beta'	gi|16272458	HI_0514	157	CP	–	–	–	–	–	111-48
HIT-like protein	gi|30995412	HI_0961	13	CP	–	–	–	–	–	111-49
Ribonuclease E	gi|30995373	HI_0413	107	CP	–	–	–	–	–	111-50
50S ribosomal protein L1	gi|16272460	HI_0516	24	CP	–	–	–	–	–	106-53
30S ribosomal protein S2	gi|161350022	HI_0913	26	CP	–	–	–	–	–	105-55
Membrane-fusion protein	gi|16272832	HI_0894	41	IM	–	–	–	–	–	102-58
30S ribosomal protein S8	gi|16272733	HI_0792	14	CP	–	–	–	–	–	101-59
50S ribosomal protein L22	gi|16272723	HI_0782	12	CP	–	–	–	–	–	96-63
Fumarate reductase flavoprotein subunit	gi|16272776	HI_0835	66	IM	–	–	–	–	–	94-65
ATP-dependent Clp protease ATPase subunit	gi|16272799	HI_0859	96	CP	–	–	–	–	–	94-67
Pyridine nucleotide transhydrogenase	gi|16273273	HI_1363	50	IM	–	–	–	–	–	91-70
Acetate kinase	gi|16273124	HI_1204	44	CP	–	–	–	–	–	90-71
Potassium efflux protein KefA	gi|16272159	HI_0195.1	127	IM	–	–	–	–	–	90-72
Penicillin-binding protein 1A	gi|16272388	HI_0440	95	IM	–	–	–	–	–	90-73
50S ribosomal protein L23	gi|16272720	HI_0779	11	CP	–	–	–	–	–	88-75
30S ribosomal protein S18	gi|16272489	HI_0545	9	CP	–	–	–	–	–	83-80
50S ribosomal protein L16	gi|16272725	HI_0784	15	CP	–	–	–	–	–	82-81
ATPase Mrp	gi|30995435	HI_1277	40	U	–	–	–	–	–	81-83
Ferric uptake regulation protein	gi|16272154	HI_0190	17	CP	–	–	–	–	–	81-84
50S ribosomal protein L4	gi|16272719	HI_0778	22	CP	–	–	–	–	–	81-85
30S ribosomal protein S16	gi|16272167	HI_0204	9	CP	–	–	–	–	–	80-86
NADH dehydrogenase	gi|16272688	HI_0747	49	IM	–	–	–	–	–	80-88
Alanyl-tRNA synthetase	gi|16272755	HI_0814	97	CP	–	–	–	–	–	79-89
50S ribosomal protein L3	gi|16272718	HI_0777	22	CP	–	–	–	–	–	78-90
Lipoprotein B	gi|16272646	HI_0706	43	OM	–	–	–	–	–	78-91
50S ribosomal protein L10	gi|16272583	HI_0640	18	CP	–	–	–	–	–	77-92
GTP-binding protein EngA	gi|16272103	HI_0136	56	CP	–	–	–	–	–	76-93
Colicin uptake protein TolR	gi|16272332	HI_0384	15	IM	–	–	–	–	–	73-97

Putative peptidase B	gi|17865546	HI_0875	47	CP	–	–	–	–	–	72-98
Phosphoglycerate kinase	gi|16272469	HI_0525	41	CP	–	–	–	–	–	72-99
Rare lipoprotein B	gi|30995409	HI_0922	19	OM	–	–	–	–	–	72-100
DL-methionine transporter ATP-binding protein	gi|16272563	HI_0621	38	IM	–	–	–	–	–	71-101
Opacity associated protein	gi|16272282	HI_0330	47	EC	–	–	–	–	–	68-102
50S ribosomal protein L20	gi|16273230	HI_1320	13	CP	–	–	–	–	–	68-103
Hypothetical protein HI1168	gi|16273092	HI_1168	14	CP	–	–	–	–	–	67-104
50S ribosomal protein L15	gi|16272738	HI_0797	15	CP	–	–	–	–	–	67-105
TonB	gi|16272209	HI_0251	29	U	–	–	–	–	–	67-107
Hypothetical protein HI0696	gi|16272638	HI_0696	141	OM	–	–	–	–	–	64-110
DNA-directed RNA polymerase subunit alpha	gi|16272744	HI_0802	36	CP	–	–	–	–	–	63-111
ATP-dependent protease ATP-binding subunit HslU	gi|16272441	HI_0497	49	CP	–	–	–	–	–	63-113
Uridine phosphorylase	gi|30995365	HI_0280	27	CP	–	–	–	–	–	62-114
Type II restriction endonuclease	gi|16273303	HI_1393	35	CP	–	–	–	–	–	61-115
cAMP-regulatory protein	gi|16272895	HI_0957	25	CP	–	–	–	–	–	61-116

[*]Identified proteins are ordered by their ion scores based on their first identification in density gradient purified preparations of Rd KW20 OMV followed by Rd Δ*vacJ* OMV, Rd Δ*yrbE* OMV, Rd KW20 OM, Rd Δ*vacJ* OM, and Rd Δ*yrbE* OM.

[†]Computed molecular protein masses in kDa according to ProtParam (http://web.expasy.org/protparam/).

[‡]Predicted subcellular protein localization according to PSORTb v3.0.2 (http://www.psort.org/psortb/). Abbreviations were used as follows: EC, extracellular; OM, outer membrane; PP, periplasmic; IM, inner membrane; CP, cytoplasmic; U, unknown.

[§]Results are given by the ion score and the ranking number of an identified protein within a respective preparation separated by a hyphen. A single dash indicates that the given protein either could not be detected in the respective preparation or showed an ion score below the identity threshold and was thus considered as not significant.

Supplementary Table 3: Bacterial strains and plasmids used in this study.

Strain or plasmid	Relevant characteristic	Reference
***H. influenzae* strains**		
Rd KW20	Unencapsulated variant of a former capsular serotype d strain, obtained from A. Wright	9
Rd AK01	Point mutation (*sxy-1*) of *tfoX* in Rd KW20, Sm^r	10
Rd Δ*vacJ*	Deletion of *vacJ* (HI_0718) in Rd KW20, Cm^r	This study
Rd Δ*yrbE*	Deletion of *yrbE* (HI_1086) in Rd KW20, Cm^r	This study
Rd Δ*fur*	Deletion of *fur* (HI_0190) in Rd KW20, Cm^r	This study
Rd Δ*vacJ*::*vacJ*	*In cis* complementation of *vacJ* (HI_0718) in Rd Δ*vacJ*, Km^r	This study
Rd Δ*yrbE*::*yrbE*	*In cis* complementation of *yrbE* (HI_1086) in Rd Δ*yrbE*, Km^r	This study
NTHi 1479-R	Spontaneous Sm^r derivative of NTHi 1479	11
NTHi 2019-R	Spontaneous Sm^r derivative of NTHi 2019	11
***V. cholerae* strains**		
Vc AC53	Spontaneous Sm^r derivative of E7946 (O1 El Tor Ogawa, clinical isolate from Bahrain 1978)	12
Vc Δ*vacJ*	Deletion of *vacJ* (VC_2048) in Vc AC53, Sm^r	This study
Vc Δ*yrbE*	Deletion of *yrbE* (VC_2519) in Vc AC53, Sm^r	This study
Vc Δ*fur*	Deletion of *fur* (VC_2106) in Vc AC53, Sm^r	This study
***E. coli* strains**		
DH5αλpir	F− *endA1 glnV44 thi-1 recA1 relA1 gyrA96 deoR nupG* Φ80d*lacZ*_M15_(*lacZYAargF*) U169, *hsdR17*(rK− mK+), *λpir*RK6, Ap^r	13
SM10λpir	*thi thr leu tonA lacY supE recA*::RPA-2-Te::Mu *λpir*R6K, Km^r	14
Ec BW	*E. coli* K12 BW25113; *rrnB3* Δ*lacZ4787 hsdR514* Δ*(araBAD)567* Δ*(rhaBAD)568 rph-1*	15
Ec Δ*fur*	Deletion of *fur* (ECK_0671) in Ec BW, Km^r	15
**Plasmids**		
pAKcat	pACYC177, Tn*10d-cat*, Km^r, Ap^r	16
pACYC177	*npt*, Km^r, Ap^r	17
pCVD442	*oriR6K mobRP4 sacB*, Ap^r	18
pCVDΔ*vacJ*	Suicide plasmid for construction of Vc Δ*vacJ*, pCVD442::Δ*vacJ*, Ap^r	This study
pCVDΔ*yrbE*	Suicide plasmid for construction of Vc Δ*yrbE*, pCVD442::Δ*yrbE*, Ap^r	This study
pCVDΔ*fur*	Suicide plasmid for construction of Vc Δ*fur*, pCVD442::Δ*fur*, Ap^r	This study
pBAD	pBAD24, arabinose-inducible, Ap^r	19
pBADmlaF-B	Expression plasmid with *mlaF*, *E*, *D*, *C*, and *B* (b_3195 to b_3191) in pBAD24, Ap^r	This study
p	pMMB67EH, IncQ broad-host-range low-copy-number cloning vector, IPTG-inducible, Ap^r	20
pvacJ	Expression plasmid with *vacJ* (VC_2048) in pMMB67EH, Ap^r	This study
pyrbE	Expression plasmid with *yrbE* (VC_2519) in pMMB67EH, Ap^r	This study

Supplementary Table 4: Oligonucleotide primers used in this study.

Usage	Oligonucleotide	Sequence (5' to 3')*
**Transposon mutagenesis**	USS+	N6AAAGTGCGGT
	USS-	N7ACCGCACTT
	blainv	CCGTAAGATGCTTTTCTGTGACTGGT
	blarev	AGTCAGGCAACTATGGATGAACGAAAT
	IS10seq_blainv	CAACTGATCTTCAGCATCTTTTAC
	IS10seq_blarev	TGCCTCACTGATTAAGCATTGGTA
**Construction of Rd Δ*vacJ***	HI0718_up_fw	GGGGTAACAATGATTGCTTCTC
	HI0718_up_rv	**TGTATGATGGTGTTTTTGAGGTG**TTTTAATCCTTACATAAATATGGGAT
	HI0718_cat_fw	**ATCCCATATTTATGTAAGGATTAAAA**CACCTCAAAAACACCATCATACA
	HI0718_cat_rv	**AATTTTTACCGCACTTTTCTGTTTA**AGGCGTTTAAGGGCACCAATAA
	HI0718_down_fw	**TTATTGGTGCCCTTAAACGCCT**TAAACAGAAAAGTGCGGTAAAAATT
	HI0718_down_rv	TCCATCTACAGAACGTAATGCC
**Construction of Rd Δ*yrbE***	HI1086_up_fw	ATAAACGAGATATTAATGAATCAAAAT
	HI1086_up_rv	**TGTATGATGGTGTTTTTGAGGTG**GATCATTCAAACAATTCCTTCACA
	HI1086_cat_fw	**TGTGAAGGAATTGTTTGAATGATC**CACCTCAAAAACACCATCATACA
	HI1086_cat_rv	**TTGTTTGTCTCATAAAAAAATCCTTA**AGGCGTTTAAGGGCACCAATAA
	HI1086_down_fw	**TTATTGGTGCCCTTAAACGCCT**TAAGGATTTTTTTATGAGACAAACAA
	HI1086_down_rv	GCAGAGGTTGATTTGTAGTATGA
**Construction of Rd Δ*fur***	HI0190_up_fw	ATGGTAGTGATACTGGAA
	HI0190_up_rv	**TGTATGATGGTGTTTTTGAGGTG**AATTTTCCTTCCTATTAT
	HI0190_cat_fw	**ATAATAGGAAGGAAAATT**CACCTCAAAAACACCATCATACA
	HI0190_cat_rv	**GAATATAAGAAAGCGACATTTTGTCGCCTTAGA**GTTTAAGGGCACCAATAACTGC
	HI0190_down_fw	**GCAGTTATTGGTGCCCTTAAAC**TCTAAGGCGACAAAATGTCGCTTTCTTATATTC
	HI0190_down_rv	TGAATTAGAACTTTCTGATGCACA
**Construction of Rd Δ*vacJ*::*vacJ***	vacJ_compl_up_fw	GGGGTAACAATGATTGCTTCTC
	vacJ_compl_up_rv	**TCTTGTGCAATGTAACATCAGAG**TTTCTCCTTAGTTGATAAAAAATTG
	vacJ_compl_npt_fw	**TTTTATCAACTAAGGAGAAA**CTCTGATGTTACATTGCACAAGA
	vacJ_compl_npt_rv	**AATTTTTACCGCACTTTTCTGTTTA**CGTGAAGAAGGTGTTGCTGAC
	vacJ_compl_down_fw	**GTCAGCAACACCTTCTTCACG**TAAACAGAAAAGTGCGGTAAAAATT
	vacJ_compl_down_rv	TCCATCTACAGAACGTAATGCC
**Construction of Rd Δ*yrbE*::*yrbE***	yrbE_compl_up_fw	ATAAACGAGATATTAATGAATCAAAAT
	yrbE_compl_up_rv	**TCTTGTGCAATGTAACATCAGAG**TTAACCTGCGCCAAACATAATAG
	yrbE_compl_npt_fw	**CTATTATGTTTGGCGCAGGTTAA**CTCTGATGTTACATTGCACAAGA
	yrbE_compl_npt_rv	**TAATTGTTTGTCTCATAAAAAAATCC**TTAGAAAAACTCATCGAGCATCAAA
	yrbE_compl_down_fw	**TTTGATGCTCGATGAGTTTTTCTAA**GGATTTTTTTATGAGACAAACAATTA
	yrbE_compl_down_rv	GCAGAGGTTGATTTGTAGTATGA
**Validation of correct construction**	HI0718_test_fw	CTGAAGCGCGTCAAACAAC
	HI1086_test_fw	GGTCATATAAAACTGATTGAAC
	HI0190_test_fw	ATGGCAATAGTTGGTCT
	cat_test_rv	GCGTTTAAGGGCACCAATAA
	npt_test_rv	AACCGTTATTCATTCGTGATTGC
**Construction of Vc Δ*vacJ***	VC2048_XbaI_up_fw	**AAATCTA**GACTGCATTATCAGCCTG
	VC2048_EcoRI_up_rv	**AAAGAATTC**CAAAGGCCGATGATCCCA
	VC2048_EcoRI_down_fw	**AATGAATTC**TGATGGTCTCTGAAATCA
	VC2048_SacI_down_rv	**AAAGAGCTC**TTTAATATTTTTTGTGAATGGATG
**Construction of Vc Δ*yrbE***	VC2519_XbaI_up_fw	**AAATCTA**GACTTAGTCACCATCAAAAATT
	VC2519_EcoRI_up_rv	**AAAGAATTC**CACATCATTTAGCACTAAACA
	VC2519_EcoRI_down_fw	**AAAGAATTC**ATGTTTGGGAACTGATCATGC
	VC2519_SacI_down_rv	**AAAGAG**CTCCGCCACATCTTCACGTT
**Construction of Vc Δ*fur***	VC2106_SacI_up_fw	**TTAGAGCTC**ACCTCCGAAACCACTTCGC
	VC2106_SalI_up_rv	**ATAGTCGAC**ATACTTTCCTGTTGATGTTCTG
	VC2106_SalI_down_fw	**AAAGTCGAC**CCATAGGCTTTACGCTCTGT
	VC2106_XbaI_down_rv	**TTATCTAGA**ACTTCCGTTACGACTACGAC
**Construction of pvacJ**	VC2048_compl_EcoRI_fw	**ATCGTTGAATTC**CAATAAGGCCGATGGG
	VC2048_compl_XbaI_rv	**GTTCTAG**ATCATAACCCTTCATTTAAATA
**Construction of pyrbE**	VC2519_compl_EcoRI_fw	**TAGGATGAATTC**TAGTGCTAAATGATGTG
	VC2519_compl_XbaI_rv	**AATCTA**GATCAGTTCCCAAACATCAATG
**Construction of pBADmlaF-B**	mlaF_EcoRI_fw	**AAAGAATTC**GAGCAGTCTGTGGCGAATTTA
	mlaB_XbaI_rv	**AAATCTAGA**TTAACGAGGCAGAACATCAGC
**Quantitative real-time RT-PCR†**	HI_rpoB-F	CGGTTTAGTGCGTGTTGAAAG
	HI_rpoB-R	TAAGTCTTGTGGCGTGATCG
	HI_vacJ-F	TCCGTGGGCATTAGTGAAAT
	HI_vacJ-R	AATTCTGCATTATTGAGATTTTTCG
	HI_yrbE-F	TCGTGTTAATCGATTTTTCTGC
	HI_yrbE-R	CAGGGCCTAATTCTCGTAAAAG

HI_fabB-F	CTAGAGGTGTAAAGGCGATTGG
HI_fabB-R	GGAGTAGCTAAACAGGCAGAAA
HI_fabD-F	AGCTGCTTCAGTCGCTATTT
HI_fabD-R	GCTATGACCTGCCATCACTT
HI_fabH-F	CTGTTGTGCTATTTGGTGATGG
HI_fabH-R	GCGTGTAAATGGGTGGAGATA
VC_rpoB-F	CTGTCTCAAGCCGGTTACAA
VC_rpoB-R	TTTCTACCAGTGCAGAGATGC
VC_vacJ-F	AGTTTCGCAAGAGGCCTTATTA
VC_vacJ-R	AGCTTGCCGTTGGAGATAAG
VC_yrbE-F	GTTCGCCAGTTATACAGCATTG
VC_yrbE-R	CTGAGCACCATGCCAATAAAG
EC_rpoB-F	AACTGCGTCTGGTGATCTATG
EC_rpoB-R	GCCCATGTAGACTTCTTGTTCT
EC_vacJ-F	GGTGATATGGCGGATGGTTT
EC_vacJ-R	TCCCTTCAAGCGTCCATTTAC
EC_yrbE-F	GGTGCGCCAGCTCTATAAT
EC_yrbE-R	CAACACCATTCCGATGAACAC

*Non-complementary nucleotides are given in bold letters and restriction sites are underlined.

†Oligonucleotide names designated with HI, VC, or EC refer to primers for *H. influenzae*, *V. cholerae*, or *E. coli*, respectively.

SUPPLEMENTARY REFERENCES

1. Reese, M.G. Application of a time-delay neural network to promoter annotation in the *Drosophila melanogaster* genome. *Comput. Chem.* **26**, 51-56 (2001).
2. Rice, P., Longden, I. & Bleasby, A. EMBOSS: the European Molecular Biology Open Software Suite. *Trends Genet.* **16**, 276-277 (2000).
3. Li, W. *et al.* The EMBL-EBI bioinformatics web and programmatic tools framework. *Nucleic Acids Res.* **43**, W580-584 (2015).
4. Goujon, M. *et al.* A new bioinformatics analysis tools framework at EMBL-EBI. *Nucleic Acids Res.* **38**, W695-699 (2010).
5. McWilliam, H. *et al.* Analysis Tool Web Services from the EMBL-EBI. *Nucleic Acids Res.* **41**, W597-600 (2013).
6. Harrison, A. *et al.* Ferric uptake regulator and its role in the pathogenesis of nontypeable *Haemophilus influenzae*. *Infect. Immun.* **81**, 1221-1233 (2013).
7. Escolar, L., Perez-Martin, J. & de Lorenzo, V. Opening the iron box: transcriptional metalloregulation by the Fur protein. *J. Bacteriol.* **181**, 6223-6229 (1999).
8. Mey, A.R., Wyckoff, E.E., Kanukurthy, V., Fisher, C.R. & Payne, S.M. Iron and Fur regulation in *Vibrio cholerae* and the role of Fur in virulence. *Infect. Immun.* **73**, 8167-8178 (2005).
9. Wilcox, K.W. & Smith, H.O. Isolation and characterization of mutants of *Haemophilus influenzae* deficient in an adenosine 5'-triphosphate-dependent deoxyribonuclease activity. *J. Bacteriol.* **122**, 443-453 (1975).
10. Herbert, M., Kraiss, A., Hilpert, A.K., Schlor, S. & Reidl, J. Aerobic growth deficient *Haemophilus influenzae* mutants are non-virulent: implications on metabolism. *Int. J. Med. Microbiol.* **293**, 145-152 (2003).
11. Roier, S. *et al.* Intranasal immunization with nontypeable *Haemophilus influenzae* outer membrane vesicles induces cross-protective immunity in mice. *PLoS One* **7**, e42664 (2012).

12. Miller, V.L., DiRita, V.J. & Mekalanos, J.J. Identification of *toxS*, a regulatory gene whose product enhances *toxR*-mediated activation of the cholera toxin promoter. *J. Bacteriol.* **171**, 1288-1293 (1989).

13. Hanahan, D. Studies on transformation of *Escherichia coli* with plasmids. *J. Mol. Biol.* **166**, 557-580 (1983).

14. Miller, V.L. & Mekalanos, J.J. A novel suicide vector and its use in construction of insertion mutations: osmoregulation of outer membrane proteins and virulence determinants in *Vibrio cholerae* requires toxR. *J. Bacteriol.* **170**, 2575-2583 (1988).

15. Baba, T. *et al.* Construction of *Escherichia coli* K-12 in-frame, single-gene knockout mutants: the Keio collection. *Mol. Syst. Biol.* **2**, 2006 0008 (2006).

16. Kraiss, A., Schlor, S. & Reidl, J. In vivo transposon mutagenesis in *Haemophilus influenzae*. *Appl. Environ. Microbiol.* **64**, 4697-4702 (1998).

17. Rose, R.E. The nucleotide sequence of pACYC177. *Nucleic Acids Res.* **16**, 356 (1988).

18. Donnenberg, M.S. & Kaper, J.B. Construction of an *eae* deletion mutant of enteropathogenic *Escherichia coli* by using a positive-selection suicide vector. *Infect. Immun.* **59**, 4310-4317 (1991).

19. Guzman, L.M., Belin, D., Carson, M.J. & Beckwith, J. Tight regulation, modulation, and high-level expression by vectors containing the arabinose pBAD promoter. *J. Bacteriol.* **177**, 4121-4130 (1995).

20. Morales, V.M., Backman, A. & Bagdasarian, M. A series of wide-host-range low-copy-number vectors that allow direct screening for recombinants. *Gene* **97**, 39-47 (1991).
